# Screening for metabolic-associated fatty liver disease in type 2 diabetes patients using non-invasive scores and ultrasound: a cross-sectional study in Egypt

**DOI:** 10.1186/s12876-025-03844-9

**Published:** 2025-09-15

**Authors:** Atteyat A. Semeya, Raafat S. A. Abdel Hafez, Suzan H. M. Ewais, Sahar M. Mostafa, Ahmed Eldeeb, Rasha Elgamal, Amira A. A. Othman

**Affiliations:** 1 Gastroenterology and Infectious Diseases Department, Benha Teaching Hospital, Benha, El-Qalyubia Egypt; 2 Internal Medicine Department, Damanhour Teaching Hospital, Damanhour, El-Beheira, Egypt; 3 Gastroenterology, Hepatology and Endemic Medicine Department, National Hepatology and Tropical Medicine Research Institute, Cairo, Egypt; 4 Clinical Pathology Department, Faculty of Medicine, Suez University, Suez, Egypt; 5 Internal Medicine Department, Faculty of Medicine, Suez University, Suez, 43511 Egypt

**Keywords:** Metabolic-Associated Fatty Liver Disease, Type 2 Diabetes Mellitus, FIB-4 Score, Liver Enzymes, Ultrasound, Non-invasive Indices

## Abstract

**Background:**

MAFLD is very common among T2DM patients and contributes significantly to both liver and systemic complications. This study aimed to evaluate the reliability of non-invasive scores and abdominal ultrasound for diagnosing and screening MAFLD in Egyptian T2DM patients.

**Methods:**

A cross-sectional study was conducted on 300 patients with T2DM who attended the Diabetes Outpatient Clinic at Benha Teaching Hospital. Liver enzymes, non-invasive fibrosis (FIB-4 and NFS), and steatosis (HSI and FLI) indices were evaluated alongside abdominal ultrasonography. Patients were stratified into two groups based on MAFLD diagnosis and assessed for disease presence and severity predictors using logistic regression models.

**Results:**

MAFLD prevalence was 46.33%. FIB-4 with an AUC of 0.826 (95% CI:0.778–0.875)and NFS with an AUC of 0.964 (95% CI:0.942–0.986) demonstrated high diagnostic accuracy for fibrosis, while HSI with an AUC of 0.847 (95% CI:0.803–0.890) and FLI with an AUC of 0.835 (95% CI:0.789–0.881) effectively identified hepatic steatosis. The HSI (38.31 ± 6.93) and FLI (68.78 ± 29.98) placed patients in the high probability category for liver steatosis, while the FIB-4 (1.94 ± 0.81) and NFS (0.56 ± 1.24) scores indicated moderate fibrosis risk. Ultrasound findings corroborated these results, with 80.58% of patients presenting with mild to moderate steatosis. Higher BMI, increased waist circumference, elevated liver function markers (elevated ALT, AST, GGT, and albumin), higher lipid profile, and poor glycemic control (HbA1c) were key predictors of MAFLD.

**Conclusions:**

Non-invasive indices alongside ultrasound support screening efforts to detect MAFLD in T2DM patients early, offering opportunities for timely management and prevention of disease progression.

## Introduction

The nomenclature MAFLD was changed to more accurately reflect the pathogenesis and cardiometabolic implications of this prevalent liver illness, which was formerly known as non-alcoholic fatty liver disease. This modification departs from definitions based on exclusion criteria, such as the lack of major alcohol consumption, and recognizes metabolic dysfunction as the main cause of hepatic steatosis [1]. In line with a patient-centered approach to diagnosis and treatment, the revised language improves clinical and research clarity [2]. With no recognized medication, MAFLD has become the most prevalent chronic liver disease in the world, affecting almost one-third of adults worldwide and posing a serious health and financial burden [3]. Numerous causes, such as sedentary lives, poor levels of physical activity, excessive calorie intake, and unsuitable diets, have contributed to its rising prevalence. Even people of average weight frequently have poor metabolic health, particularly in wealthy nations. There is an urgent need for improved definitions and diagnostic criteria since the lack of a clear clinical definition of diagnosis and standard nomenclature is a significant obstacle in controlling MAFLD [4].

According to recent statistics on death and disability rates from the Global Burden of Disease (GBD) research, the health and economic burden of MAFLD is seriously rising in many regions of the world [5]. In addition to potentially improving research goals and resource allocation, it is hoped that the changes in nomenclature and the emphasis on metabolic dysfunction would improve the way we view, diagnose, and treat this prevalent liver illness. More than 96% of people with NAFLD match the requirements for MAFLD, according to emerging data, indicating an excellent concordance rate between the two classifications. So, both terms can be used interchangeably [6]. A wide variety of progressive steatotic liver diseases are included in MAFLD, from solitary hepatic steatosis to metabolic dysfunction-associated steatohepatitis (MASH), which can lead to variable degrees of liver fibrosis in addition to an increased risk of liver-related complications, including cirrhosis, end-stage liver disease, and hepatocellular carcinoma (HCC) [7, 8]. Indeed, MAFLD is linked to a higher risk of developing several extrahepatic manifestations, including cardiovascular disease (CVD), chronic kidney disease (CKD), and specific types of extrahepatic cancers [9].

The screening for MAFLD is very important in the early stages of detection and prevention of liver-related complications such as liver fibrosis and cirrhosis, especially in individuals presenting with metabolic risk factors such as obesity, type 2 diabetes, and metabolic syndrome [10]. Non-invasive diagnostic and screening methods have gained popularity due to their safety and ease, such as non-invasive diagnostic indices (e.g. FIB-4, NFS, HSI, and FLI scores) [11], clinical and laboratory data, including age, platelet count, ALT, and AST, determines the risk of fibrosis [12–14]. Abdominal ultrasonography is another imaging modality that is widely accessible and reasonably priced, although it has a low sensitivity for detecting early stages of hepatic steatosis [15], thus affording the healthcare provider a means to assess risk without dependence on invasive procedures such as liver biopsy despite being the gold standard in the diagnosis [16]. Regular screening among the at-risk population is very important, since early diagnosis may result in interventions that limit disease progression and enhance long-term outcomes.

Globally, fatty liver disease affects approximately 30.2% of the population, with higher prevalence observed in individuals with metabolic syndrome and T2DM [17]. This highlights the substantial burden of MAFLD, particularly in high-risk populations such as patients with T2DM in Egypt.

For population-wide screening and monitoring of MAFLD, non-invasive methods are becoming more and more popular, particularly among high-risk populations such as those with obesity and type 2 diabetes. These techniques might make it easier to identify those who are at risk early on, enabling prompt interventions and reducing the need for intrusive procedures. Our study aimed to screen T2DM patients for MAFLD using liver enzymes, FIB-4 index, and pelvic-abdominal ultrasound, and to evaluate the reliability of these tools in detecting liver involvement.

## Patients and methods

### Study population and design

A cross-sectional study was conducted on 300 Type 2 Diabetes Mellitus (T2DM) patients attending the Diabetes Outpatient Clinic of Benha Teaching Hospital. Participants were consecutively enrolled from the outpatient registry between December 2022—2023. Two blinded endocrinologists independently verified eligibility (κ = 0.91). The Cochran formula for prevalence studies was adopted in the determination of the sample size for the current study. The Z-score reflecting a 95% confidence level with an alpha level of 0.05 is 1.96. Based on these and taking the previously reported prevalence as 20% among Type 2 Diabetes Mellitus patients with fatty liver disease [18], the minimum number of respondents to be included according to the calculation is approximately 246 participants. A sample size of 300 participants was agreed upon to get robust data and ensure statistical power.

In this study, the likelihood of MAFLD was initially assessed based on metabolic dysfunction, specifically the presence of T2DM, obesity (BMI ≥ 30 kg/m^2^), or increased waist circumference (≥ 102 cm for men, ≥ 88 cm for women). Fatty liver disease was definitively diagnosed using abdominal ultrasound scans to detect hepatic steatosis, combined with metabolic criteria such as T2DM or obesity. After confirming the diagnosis of MAFLD, the subjects were divided into two groups: Group 1 (DM with MAFLD): 139 diabetic patients who fulfilled the diagnostic criteria of MAFLD based on imaging findings along with raised liver enzyme levels; and Group 2 (DM without MAFLD): 161 diabetic patients without any evidence of fatty liver disease as per imaging and metabolic dysfunction criteria.

### Ethical considerations

The current study was implemented in coordination with the guidelines of the Declaration of Helsinki. Ethical approval was gained according to the recommendations of the Ethics Unit of Benha Teaching Hospitals, Egypt, Approval #: HB-000121. Informed consent was obtained from the patients, who were informed of all the steps of the study and their right to withdraw at any time.

### Inclusion criteria

The inclusion criteria were the diagnosis of Type 2 Diabetes Mellitus, aged 18 years and above, with no history of hepatic diseases such as cirrhosis, hepatitis, or liver cancer. They also had no history of malignancies or other serious systemic illnesses that might interfere with the study outcomes and were willing to participate in the study and provide informed written consent. Moreover, all the subjects underwent blood sampling for the assessment of liver enzymes, as well as abdominal ultrasound.

### Exclusion criteria

The following conditions were excluded from the study: Type 1 Diabetes Mellitus, as the present study focused on Type 2 diabetes; known history of any liver diseases: cirrhosis, hepatitis, liver cancer, and malignant diseases in history, pregnancy or lactation; and patients with prior liver transplantation or other major hepatobiliary interventions (e.g., liver resection, transjugular intrahepatic portosystemic shunt [TIPS]). Other exclusions included a history of alcohol abuse or conditions leading to secondary causes of fatty liver, chronic kidney disease with significantly impaired renal function, and anyone unable or unwilling to give informed consent.

### Clinical assessment

#### Demographic and clinical evaluation

All subjects were subjected to full history taking, and thorough physical examination. Demographic and clinical data, including age, sex, and medical history, were recorded.

#### Anthropometric measurements

In all participants, weight and height were measured in light clothes and without shoes. Waist circumference (WC) was also measured at the midpoint between the lower costal margin and the iliac crest to the nearest 0.1 cm. Body weight was measured to the nearest 0.1 kg and height to the nearest 0.1 cm. Using these measures, body mass index (BMI) was calculated by the formula: BMI = Weight (kg) / Height (m)^2^.

#### Abdominal ultrasonography

Real-time abdominal ultrasonography was performed using a Trans-Abdominal 4C-AH46701AA apparatus [19]. The examination evaluated for the diagnosis of fatty liver diseases as per the standard criteria of the American Gastroenterology Association's criteria for diagnosis [20], which include hepatic echogenicity, enhancement, and vascular wall differentiation. Evaluation of the liver was done for size, echo pattern, and the presence of hepatic focal lesions. Other studies included spleen evaluation, ascites detection, abdominal lymphadenopathy, and scanning of other abdominal and pelvic organs. Hepatic steatosis was diagnosed and graded using ultrasonographic findings during the diagnostic evaluation. Steatosis was considered “ absent (Grade 0) in the presence of normal liver echotexture; mild (Grade 1) when a slight and diffuse increase in liver echogenicity was present with normal visualization of the diaphragm and portal vein wall; moderate (Grade 2) in the presence of a moderate increase in liver echogenicity, with slightly impaired visualization of the portal vein wall and diaphragm; severe (Grade 3) in cases of a marked increase in liver echogenicity, with poor or no visualization of the portal vein wall, diaphragm, and posterior part of right liver lobe”. This gave a uniform assessment of the severity of hepatic steatosis in the study participants [21].

#### Laboratory investigations

Five milliliters (mL) of fresh venous blood were collected from each participant after an overnight fast, with 2.5 mL of ethylenediaminetetraacetic acid (EDTA) for complete blood count (CBC) and glycated hemoglobin (HbA1c), and 2.5 mL drawn without anticoagulants for other serum testing. The samples were then centrifuged at 3000 rpm for 15 min, and serum aliquots were stored at -20 °C until further analysis. All samples were promptly sent to the laboratory, where serum tests for lipid profile (total cholesterol, triglycerides, low-density lipoprotein, high-density lipoprotein), liver function (alanine aminotransferase, aspartate aminotransferase), and kidney function (creatinine, urea) using the automated Cobas c 111 analyzers (Roche Diagnostics). The CBC and HbA1c were analyzed using the Sysmex XN-550 Cell Counter. The commonly used biochemical thresholds for diagnosing MAFLD or abnormal liver function for Alanine Aminotransferase (ALT) is 25 U/L for males and 17 U/L for females. Aspartate Aminotransferase (AST) is often considered abnormal if > 40 U/L [22, 23].

#### Calculation of fibrosis indices

Calculation of Fibrosis-4 score (FIB-4) by the following formula [24]:

$$\mathbf{FIB}\boldsymbol-\mathbf4\boldsymbol\;\mathbf{index}\boldsymbol=\frac{\mathbf{Age}\boldsymbol\;\boldsymbol(\mathbf{years}\boldsymbol)\boldsymbol\times\mathbf{AST}\mathbf{\left({U/L}\right)}}{\mathbf{Platelet}\boldsymbol\;\mathbf{count}\mathbf{\left({10^9/L}\right)}\boldsymbol\times\sqrt{\mathbf{ALT}\mathbf{\left({U/L}\right)}}}$$

#### Interpreting FIB-4 score

The FIB-4 index helps in stratifying the patients into various groups of risk regarding liver fibrosis. A FIB-4 score of < 1.45 is taken to indicate a low risk for significant fibrosis, suggesting a low likelihood of advanced liver disease, for which further evaluation may not be necessary. Scores of FIB-4 between 1.45 and 3.25 reflect indeterminate risk. Patients in this range will likely need additional tests confirming the extent of fibrosis, such as liver imaging e.g., elastography or FibroScan or liver biopsy. The FIB-4 score of > 3.25 signifies a high risk for significant fibrosis or cirrhosis, where a high likelihood of advanced fibrosis is considered; thus, further management, including referral for liver biopsy or advanced imaging, is recommended [25].

Calculation of NAFLD fibrosis score (NFS) by the following formula [26]:

$$\mathrm{NFS}=-1.675+0.037\times\mathrm{Age}(\mathrm{years})+0.094\times\mathrm{BMI}(\mathrm{kg}/\mathrm m^2)+1.13\times\mathrm{IFG}/\mathrm{diabetes}\left(\mathrm{yes}=1,\mathrm{no}=0\right)+0.99\times\mathrm{AST}/\mathrm{ALTratio}-0.013\times\mathrm{Plateletcount}(\times10^9)-0.66\times\mathrm{Albumin}(\mathrm g/\mathrm{dL})$$

#### Interpreting NFS score

The NAFLD Fibrosis Score allows stratification of the patients according to their possibility of having advanced liver fibrosis. An NFS < -1.455 means a low likelihood of advanced fibrosis, having a negative predictive value of approximately 90%, and therefore, clinically significant fibrosis is unlikely. A score ranging from -1.455 to 0.675 is an indeterminate risk, and additional evaluation may be recommended for staging the extent of fibrosis, as with FibroScan or liver biopsy. An NFS above 0.675 indicates an increased probability of advanced fibrosis and carries an approximate 82% positive predictive value for such a condition, reflecting more severe liver damage [25].

#### Calculation of steatosis indices

Calculation of Hepatic steatosis index (HSI) by the following formula [27]:

$$\mathrm{HSI}=8\times\frac{\mathrm{ALT}}{\mathrm{AST}}+\mathrm{BMI}+\left(+2\;\mathrm{if}\;\mathrm{Female}\right)+\left(+2\;\mathrm{if}\;\mathrm{Diabetes}\;\left(\mathrm{Yes}=1\right)\right)$$

#### Interpreting HSI score

The Hepatic Steatosis Index is a method of assessing the probability of hepatic steatosis. An HSI < 30 indicates a low probability of hepatic steatosis and, therefore, a low concern for fatty liver. A score between 30 and 36 is indeterminate, where confirmation of liver fat by imaging or biopsy may be required. An HSI > 36 indicates a high probability of hepatic steatosis, and this would suggest the presence of fatty liver that has to be further managed clinically [25].

Calculation of Fatty liver index (FLI) by the following Formula [28]:

The FLI is calculated using the following formula:

$$\mathrm{FLI}=\frac{e^{\left(0.953\times\ln\left(\mathrm{TG}\right)+0.139\times\mathrm{BMI}+0.718\times\ln\left(\mathrm{GGT}\right)+.053\times\mathrm{WC}-15.745\right)}}{1+e^{\left(0.953\times\ln\left(\mathrm{TG}\right)+0.139\times\mathrm{BMI}+0.718\times\ln\left(\mathrm{GGT}\right)+.053\times\mathrm{WC}-15.745\right)}}\times100$$

Where: ln = natural logarithm, TG = Triglycerides (mg/dL), BMI = Body Mass Index (kg/m^2^), GGT = Gamma-Glutamyl Transferase (U/L), WC = Waist Circumference (cm).

#### Interpreting FLI scores

The Fatty Liver Index is used to determine the probability of a fatty liver. FLI < 30: low probability of fatty liver; this suggests a minimal concern. FLI ≥ 60: high probability of fatty liver; this suggests a strong likelihood of the condition. FLI between 30 and 60: intermediate probability, in which further diagnostic evaluation may be required through imaging or biopsy to confirm the diagnosis.

These indices were selected for their clinical relevance. The fibrosis indices, FIB-4 and NFS, enable the estimation of the degree of liver fibrosis, a critical predictor of long-term liver-related outcomes in patients with metabolic diseases. Steatosis indices, HSI and FLI, reflect the degree of fat accumulation in the liver, being central to the diagnosis and monitoring of MAFLD, especially in diabetic patients. Generally, high scores in these indices reflect an increased risk for liver progression, such as cirrhosis or hepatocellular carcinoma [25].

### Statistical analysis

Statistical analysis was conducted using SPSS v26 (IBM Inc., Armonk, NY, USA). The Shapiro–Wilk test and histograms were used to assess the normality of data distribution. Quantitative parametric data were presented as mean ± standard deviation (SD) and analyzed using an unpaired Student’s t-test. Non-parametric data were expressed as median (interquartile range, IQR) and analyzed using the Mann–Whitney U test. Qualitative data were presented as frequency and percentage (%) and analyzed using the Chi-square test or Fisher's exact test when appropriate. ROC curve analysis was performed to evaluate the ability of non-invasive indices (HSI, FLI, FIB-4, NFS) to discriminate between ultrasound-diagnosed MAFLD and non-MAFLD patients. Sensitivity, specificity, and AUC values were reported with 95% confidence intervals (CIs). Missing data for key variables were addressed using multiple imputation methods. No patients were lost to follow-up, as this was a single-visit study. A two-tailed *P* ≤ 0.05 was considered statistically significant.

## Results

### Baseline characteristics

The study included 300 participants with T2DM, with a mean age of 57.37 ± 6.06 years. The cohort was nearly equally distributed by sex (female-to-male ratio: 1.05:1). Hypertension was the most common comorbidity, affecting 58% of participants. The overall prevalence of MAFLD was 46.33% (139/300).

Patients with higher BMI (*p* = 0.001) and waist circumference (*p* = 0.03) were more likely to exhibit features consistent with metabolic dysfunction, underscoring the role of obesity and visceral adiposity in the pathogenesis of MAFLD. In contrast, there were no significant differences between the two groups in terms of age, sex, smoking, or hypertension between patients suspected of having MAFLD and those without clinical suspicion (Table 1).

**Table 1:** Baseline characteristics of the study groups

**Variables**		**DM with MAFLD (*n*** **=139)**	**DM without MAFLD (*n*** **=161)**	***P*** **value**
**Age (years)**		58.04 ± 4.89	56.79 ± 6.87	0.075
**Sex**	**Male**	62 (44.60%)	84 (52.17%)	0.191
	**Female**	77 (55.40%)	77 (47.83%)	
**BMI (kg/m**^**2**^**)**		30.81 ± 5.66	28.87 ± 4.69	0.001*
**WC cm**	**Male**	104.12 ± 7.51	90.37 ± 5.12	0.030*
	**Female**	94.25 ± 6.49	80.12 ± 4.07	
**Smoking**		47 (33.81%)	49 (30.43%)	0.532
**HTN**		81 (58.27%)	93 (57.76%)	0.929

Data are presented as mean ± SD or frequency (%)

*DM* Diabetes mellitus, *MAFLD* Metabolic-associated fatty liver disease, *BMI* Body mass index, *WC* Waist Circumference, *HTN* Hypertension

^*^significant as *P value* < *0.05*

### Hematological and biochemical parameters

Hematological parameters showed no significant differences in hemoglobin (*p* = 0.096) between groups, suggesting that anemia was not influenced by MAFLD. Total leucocyte count and platelet counts were significantly higher in diabetic patients with MAFLD (*p* < 0.001 and *p* = 0.002, respectively), possibly reflecting the early MAFLD stages, where platelet counts may be normal or slightly elevated due to increased inflammatory activity (Table 2).

**Table 2:** Hematological and biochemical parameters of the study groups

Variables		DM with MAFLD (*n* = 139)	DM without MAFLD (*n* = 161)	*P* value
Hematological parameters	Hb (g/dl) TLC (× 10^9^/L) Platelets (× 10^9^/L)	10.7 ± 0.82 11.62 ± 1.71 223.28 ± 65.84	10.91 ± 1.24 5.77 ± 1.07 201.11 ± 56.82	0.096 < 0.001* 0.002*
Lipid profile	Cholesterol (mg/dl) TG (mg/dl) LDL (mg/dl) HDL (mg/dl)	220.97 ± 51.97 172.21 ± 61.05 147.19 ± 35.57 54.35 ± 12.83	180.27 ± 20.83 139.57 ± 36.44 102.42 ± 28.97 64.98 ± 11.45	< 0.001* < 0.001* < 0.001* < 0.001*
Liver Functions	ALT (U/L) AST (U/L) GGT (U/L) Albumin (g/dL)	34.45 ± 19.89 44.19 ± 18.45 80 ± 0.88 2.7 ± 0.5	29.9 ± 11.85 19.98 ± 5.81 42 ± 0.30 3.5 ± 1.4	0.015* < 0.001* < 0.001* < 0.001*
Kidney functions	Creatinine (mg/dl) Urea (mg/dl)	1.07 ± 0.45 12.68 ± 4.44	1.03 ± 0.31 12.41 ± 4.62	0.406 0.613
HbA1c (%)		7.06 ± 1.32	6.6 ± 0.71	< 0.001*

Data are presented as mean ± SD

*DM* Diabetes mellitus, *MAFLD* Metabolic-associated fatty liver disease, *Hb* Hemoglobin, *TLC* Total leucocyte count, *TG* Triglycerides, *LDL* Low-density lipoprotein, *HDL* High-density lipoprotein, *ALT* Alanine aminotransferase, *AST* Aspartate aminotransferase, *GGT* Gamma-glutamyl transferase, *HbA1c* Hemoglobin A1c

^*^significant as *P value* < *0.05*

The significantly higher HbA1c levels in MAFLD patients compared to non-MAFLD patients underscore the strong link between poor glycemic control and the development of metabolic-associated fatty liver disease because of sustained hyperglycemia, which leads to insulin resistance, a key factor in de novo lipogenesis, decreases lipid oxidation, and TG deposition in the liver, contributing to MAFLD. Optimizing blood sugar levels, especially in T2D, by regular HbA1c measurements helps with early screening and identification of MAFLD in patients with T2DM (Table 2).

The lipid profile abnormalities, particularly high LDL and TG levels and low HDL, reinforce the association between MAFLD and dyslipidemia, both of which are integral components of metabolic syndrome. These findings strongly support the use of routine biochemical tests for early screening and identification of MAFLD in patients with T2DM (Table 2).

The significantly elevated liver enzymes (ALT, AST) suggest ongoing hepatocellular injury, consistent with hepatic steatosis or early-stage fibrosis in MAFLD patients. The elevated liver enzyme (GGT) suggests increased oxidative stress and bile duct activity associated with hepatic steatosis and inflammation. The significantly elevated GGT reflects a metabolic liver dysfunction and is often linked to insulin resistance and oxidative damage, which are central to the pathophysiology of MAFLD. Albumin levels were significantly lower in MAFLD patients compared to non-MAFLD patients, suggesting an early decline in liver synthetic function despite the absence of overt liver failure. Reduced albumin may also indicate a systemic inflammatory state or subtle protein metabolism alterations in MAFLD. Low albumin levels have also been linked to increased cardiovascular risk and mortality in metabolic syndrome, making it an important marker for both liver and systemic disease severity. Integrating these markers into routine biochemical evaluations allows for a more nuanced assessment of liver health and screening for and monitoring MAFLD in patients with T2DM (Table 2).

In contrast, creatinine and urea showed no significant differences between the two groups indicating preserved renal function and no systemic inflammatory response in both groups (Table 2).

### Abdominal ultrasonography results

#### Diagnostic findings

MAFLD was diagnosed based on the presence of hepatic steatosis detected by abdominal ultrasound, combined with metabolic dysfunction criteria such as T2DM, obesity, or other components of metabolic syndrome. Among the 300 participants, 46.33% (139/300) were diagnosed with MAFLD. This prevalence highlights the significant burden of metabolic dysfunction-related liver disease in the study population.

#### Severity of steatosis by ultrasound

**Fig. 1:**
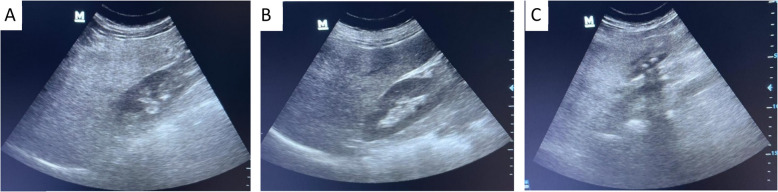
Ultrasound grading of hepatic steatosis based on liver echogenicity and visualization of key structures. **A** Grade 1: Slight and diffuse increase in liver echogenicity with normal visualization of the diaphragm and portal vein wall. **B** Grade 2: Moderate increase in echogenicity with slightly impaired visualization of the portal vein wall and diaphragm. **C** Grade 3: Marked increase in echogenicity with poor or no visualization of the portal vein wall, diaphragm, and posterior part of the right liver lobe. All three steatosis grades are represented in the figure

Abdominal ultrasonography revealed varying degrees of hepatic steatosis among the 139 MAFLD patients. Mild steatosis (grade 1) was observed in 30.94% of cases, moderate steatosis (grade 2) in 49.64%, and severe steatosis (grade 3) in 19.42%. These findings indicate that most patients presented with moderate-grade steatosis, while a considerable number exhibited mild or severe conditions. The varying severity underscores the diverse spectrum of liver involvement in MAFLD and the need for individualized management strategies tailored to the degree of hepatic damage (Fig. 1).

### Fibrosis and steatosis indices

In addition to ultrasound, non-invasive indices were utilized to further assess liver health and stratify patients based on their risk of fibrosis or steatosis. MAFLD patients exhibited significantly higher values in FIB-4, NFS, HSI, and FLI compared to those without MAFLD (all *p* < 0.001). Their utility in this study highlights the potential for integrating non-invasive tools into routine clinical practice for screening and managing patients with T2DM at risk of MAFLD.

Regarding the Fibrosis-4 score (FIB-4), NAFLD Fibrosis Score (NFS), Hepatic Steatosis Index (HSI), and Fatty Liver Index (FLI), there were significant differences between the two groups. In diabetic patients with MAFLD, the FIB-4 had a mean of 1.94 (SD = 0.81), while in diabetic patients without MAFLD, it was significantly lower with a mean of 1.04 (SD = 0.46, *p* < 0.001). Similarly, the NFS was significantly higher in diabetic patients with MAFLD, with a mean of 0.56 (SD = 1.24), compared to -1.92 (SD = 0.63, *p* < 0.001) in those without MAFLD. For the HSI, diabetic patients with MAFLD had a mean of 38.31 (SD = 6.93), significantly higher than the mean of 29.38 (SD = 5.04, *p* < 0.001) in diabetic patients without MAFLD. Lastly, the FLI showed a higher mean in the MAFLD group at 68.78 (SD = 29.98) compared to 30.77 (SD = 29.76, *p* < 0.001). These findings suggest that diabetic patients with MAFLD tend to have significantly higher values in these indices compared to those without MAFLD (Table 3).

**Table 3:** Fibrosis and steatosis indices of the study groups

Score/Index	DM with MAFLD (*n* = 139)	DM without MAFLD (*n* = 161)	*p*-value	Risk/Probability Categories [25]
FIB-4	1.94 ± 0.81	1.04 ± 0.46	< 0.001	< 1.45: Low risk; 1.45–3.25: Intermediate risk; > 3.25: High risk
NFS	0.56 ± 1.24	-1.92 ± 0.63	< 0.001	< -1.455: Low risk; -1.455 to 0.675: Indeterminate risk; > 0.675: High risk
HSI	38.31 ± 6.93	29.38 ± 5.04	< 0.001	< 30: Low probability; 30–36: Indeterminate probability; > 36: High Probability
FLI	68.78 ± 29.98	30.77 ± 29.76	< 0.001	< 30: Low probability; 30–60: Intermediate probability; ≥ 60: High Probability

*FIB-4* Fibrosis-4 score, *NFS* NAFLD Fibrosis Score, *HSI* Hepatic Steatosis Index, *FLI* Fatty Liver Index

^*^significant as *P* value < 0.05

### Diagnostic performance of fibrosis and steatosis indices

#### Sensitivity, specificity, and predictive metrics

**Fig. 2:**
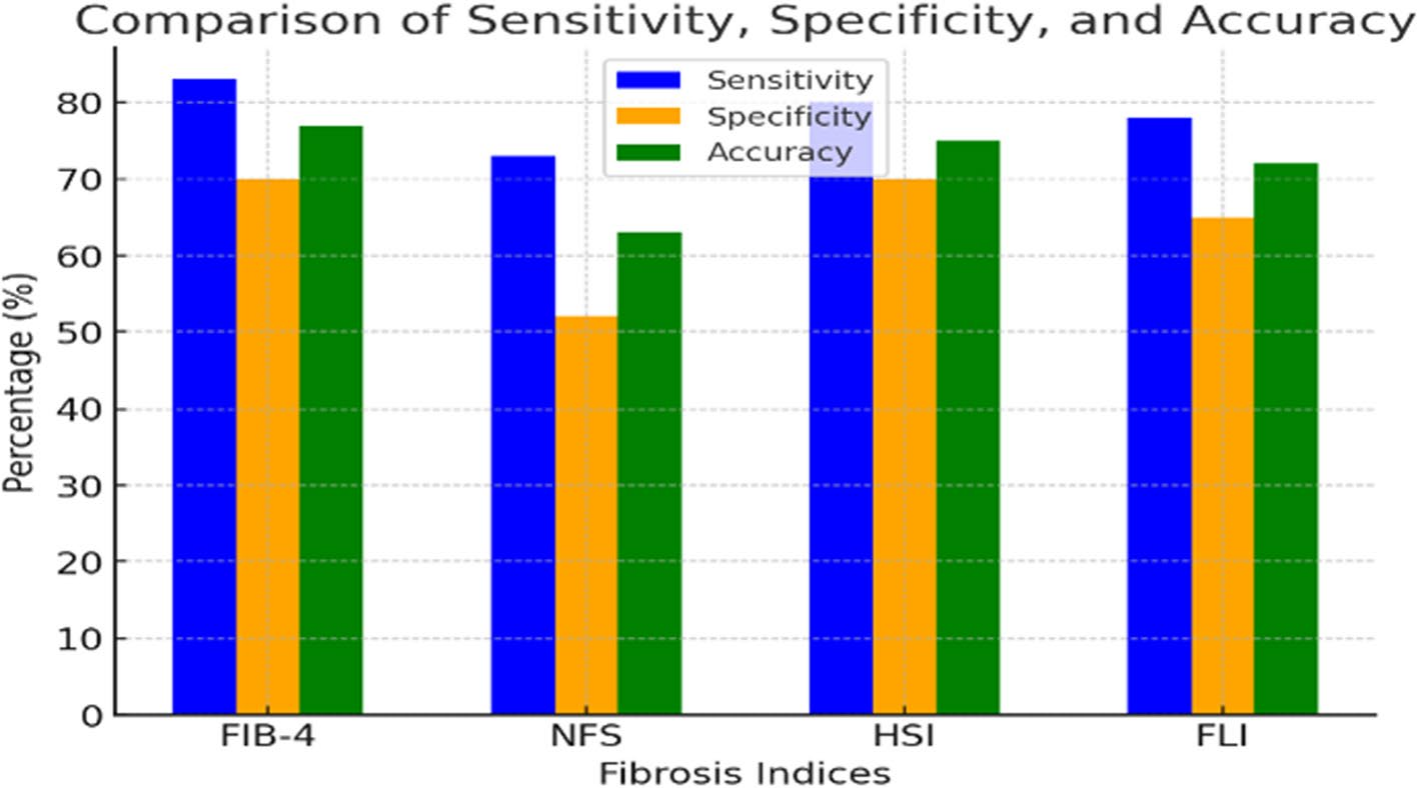
Comparison of sensitivity, specificity, and accuracy for fibrosis (FIB-4, NFS) and steatosis (HSI, FLI) indices. Cut-off values: FLI ≥ 60, HSI ≥ 36, FIB-4 > 1.45, NFS > 0.675

The sensitivity, specificity, accuracy, and likelihood ratios (LR + and LR −) of the four non-invasive indices were evaluated at their respective optimal cutoff points, highlighting their diagnostic utility in clinical practice. For FIB-4, a cutoff of 1.96 yielded a sensitivity of 83%, specificity of 70%, accuracy of 77%, LR + of 2.7, and LR − of 0.2. The NAFLD Fibrosis Score (NFS), at a cutoff of 0.56, demonstrated a sensitivity of 73%, specificity of 52%, accuracy of 63%, LR + of 1.5, and LR − of 0.5. The Hepatic Steatosis Index (HSI), with a cutoff of 38, showed a sensitivity of 80%, specificity of 70%, accuracy of 75%, LR + of 2.6, and LR − of 0.3. Finally, the Fatty Liver Index (FLI), at a cutoff of 68, achieved a sensitivity of 78%, specificity of 65%, accuracy of 72%, LR + of 2.2, and LR − of 0.4 (Fig. 2).

#### Diagnostic sccuracy and ROC analysis

**Fig. 3:**
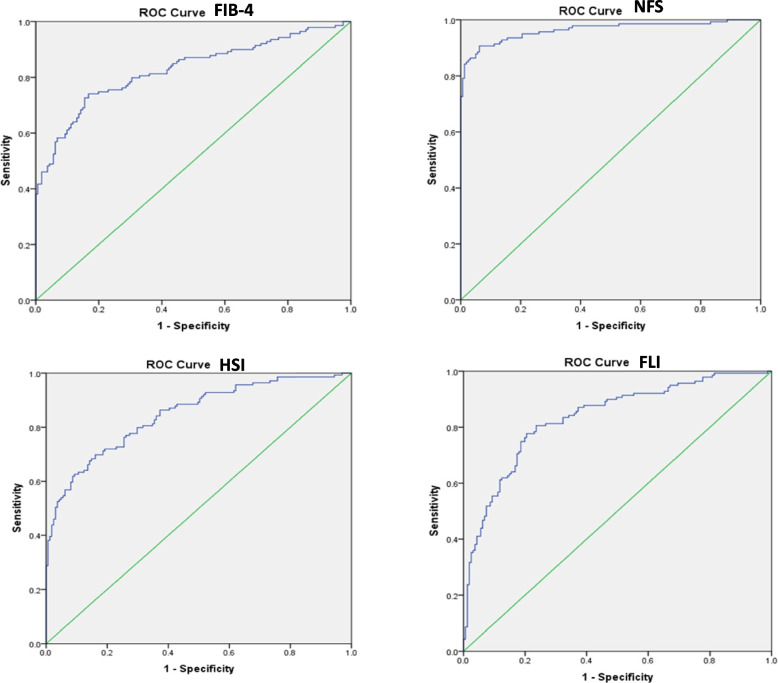
ROC curves for fibrosis (FIB-4, NFS) and steatosis (HSI, FLI) indices, demonstrating their predictive performance in ultrasound-confirmed MAFLD cases

The diagnostic performance of four non-invasive indices was assessed using receiver operating characteristic (ROC) curve analysis. Fibrosis indices, including Fibrosis-4 (FIB-4) and NAFLD Fibrosis Score (NFS), and steatosis indices, such as the Hepatic Steatosis Index (HSI) and Fatty Liver Index (FLI), were evaluated. Among the fibrosis indices, NFS demonstrated the highest diagnostic accuracy with an AUC of 0.964 (95% CI: 0.942–0.986), compared to FIB-4, which achieved an AUC of 0.826 (95% CI: 0.778–0.875). For the steatosis indices, HSI showed an AUC of 0.847 (95% CI: 0.803–0.890), while FLI achieved an AUC of 0.835 (95% CI: 0.789–0.881). These results suggest that NFS is the most reliable index for detecting fibrosis. At the same time, HSI and FLI provide comparable performance in assessing steatosis in patients with metabolic-associated fatty liver disease (MAFLD). The sensitivity, specificity, and AUC values for each index (Table 4) should be interpreted as performance against ultrasound-diagnosed fatty liver rather than an independent diagnostic accuracy measure. The ROC curves for all indices are shown in Fig. 3.

**Table 4:** Diagnostic performance of non-invasive indices for MAFLD detection

Index	Cut-off Value	Sensitivity (95% CI)	Specificity (95% CI)	Accuracy (%)	AUC (95% CI)	Source for Cut-off
HSI	≥ 36	80% (73-86)	70% (63–76)	75%	0.847 (0.803–0.890)	Lee et al., 2010 [27, 29]
FLI	≥ 60	78% (70–84)	65% (58–71)	72%	0.835 (0.789–0.881)	Bedogni et al., 2006 [28]
FIB-4	> 1.45	83% (76–88)	70% (63–76)	77%	0.826 (0.778–0.875)	Sterling et al., 2006 [24]
NFS	> 0.675	73% (66–79)	52% (45–59)	63%	0.964 (0.942–0.986)	Angulo et al., 2007 [26]

The diagnostic performance of non-invasive indices was assessed using ROC curve analysis, with ultrasound serving as the reference standard for MAFLD diagnosis (Table 4, Fig. 3). The results indicate that HSI and FLI demonstrated strong discriminative ability for hepatic steatosis, while FIB-4 and NFS showed moderate performance for fibrosis risk stratification. However, given that ultrasound has limited sensitivity for detecting mild steatosis, the reported AUC values should be interpreted as the indices' ability to approximate ultrasound-based MAFLD detection rather than biopsy-confirmed liver pathology. The cited cut-off values were derived from previous validation studies in different populations, and further research may be needed to optimize these thresholds for Egyptian cohorts.

### Predictors of MAFLD presence and severity in T2DM patients

To explore the factors associated with the development and progression of metabolic-associated fatty liver disease (MAFLD) in patients with type 2 diabetes mellitus (T2DM), binary logistic regression was performed to identify predictors of MAFLD presence and multinomial logistic regression was used to evaluate the risk of disease severity based on grades of steatosis (Grade 2 vs. Grade 1, and Grade 3 vs. Grade 1), as shown in Table 5.

**Table 5:** Predictors of MAFLD presence and severity in T2DM patients

**Predictor**	**MAFLD Presence**	**MAFLD Severity**	
		**Severity: Grade 2 vs. Grade 1**	**Severity: Grade 3 vs. Grade 1**
	**OR (95% CI) (*p*-value)**	**OR (95% CI) (*p*-value)**	**OR (95% CI) (*p*-value)**
**BMI (kg/m**^**2**^**)**	1.51 (1.25–1.82) (0.001)	1.42 (1.20–1.68) (0.001)	1.60 (1.30–1.97) (0.001)
**HbA1c (%)**	1.68 (1.34–2.12) (< 0.001)	1.28 (1.10–1.49) (0.002)	1.46 (1.21–1.75) (< 0.001)
**WC (cm)**	1.41 (1.29–1.54) (< 0.001)	1.25 (1.12–1.39) (< 0.001)	1.36 (1.21–1.53) (< 0.001)
**ALT (U/L)**	1.08 (1.02–1.14) (0.015)	1.11 (1.04–1.19) (0.004)	1.17 (1.08–1.27) (< 0.001)
**AST (U/L)**	1.25 (1.16–1.35) (< 0.001)	1.14 (1.06–1.23) (0.002)	1.25 (1.14–1.38) (< 0.001)
**GGT (U/L)**	1.21 (1.12–1.30) (< 0.001)	1.13 (1.05–1.22) (0.003)	1.21 (1.11–1.32) (< 0.001)
**Albumin (g/dL)**	0.64 (0.51–0.80) (< 0.001)	0.73 (0.60–0.89) (0.002)	0.63 (0.49–0.81) (< 0.001)
**TG (mg/dL)**	1.14 (1.08–1.21) (< 0.001)	1.10 (1.03–1.18) (0.005)	1.18 (1.10–1.27) (< 0.001)
**LDL (mg/dL)**	1.15 (1.09–1.22) (< 0.001)	1.09 (1.03–1.16) (0.005)	1.15 (1.07–1.24) (< 0.001)
**HDL (mg/dL)**	0.89 (0.85–0.94) (< 0.001)	0.92 (0.88–0.97) (0.002)	0.89 (0.84–0.94) (< 0.001)

*BMI* Body mass index, *HbA1c* Hemoglobin A1c, *WC* Waist circumference, *ALT* Alanine aminotransferase, *AST* Aspartate aminotransferase, *GGT* Gamma-glutamyl transferase, *TG* Triglycerides, *LDL* Low-density lipoprotein, *HDL* High-density lipoprotein

Predictors with *p* < 0.05 are considered statistically significant. The 95% CI indicates the range of plausible values for the odds ratio, where a CI excluding 1 confirms statistical significance

The binary logistic regression analysis revealed significant predictors for MAFLD in patients with T2DM. Of the metabolic parameters, the factors of BMI, HbA1c, and waist circumference were strongly associated with MAFLD, indicating a critical role of obesity, poor glycemic control, and visceral adiposity in the pathogenesis of the disease. Hepatic dysfunction markers also included ALT, AST, and GGT, reflecting ongoing hepatocellular injury and oxidative stress in MAFLD patients. Lower albumin levels were significantly associated with MAFLD, indicating early hepatic synthetic dysfunction and systemic metabolic disturbance. Furthermore, lipid abnormalities comprised TG, high LDL, and low HDL levels, reinforcing the integral role of dyslipidemia in the development of MAFLD (Table 5).

The multinomial logistic regression analysis showed significant predictors of MAFLD severity in T2DM patients. Positive associations were established for BMI, HbA1c, and waist circumference with both moderate and severe steatosis, thus reinforcing the role of obesity, poor glycemic control, and visceral adiposity in the disease process. Other strong predictors included hepatic markers like GGT, ALT, and albumin. Among these, high GGT and ALT represented increased oxidative stress and hepatocellular injury, while low albumin indicated a decline in liver synthetic function in severe cases. Further, the parameters of the lipid profile, including TG, high LDL, and low HDL, related to increasing severity and reflected worsening dyslipidemia as steatosis progressed (Table 5).

Multivariate logistic regression identified BMI (OR: 1.51, 95% CI: 1.25–1.82, *p* = 0.001), HbA1c (OR: 1.68, 95% CI: 1.34–2.12, *p* < 0.001), and triglyceride levels (OR: 1.14, 95% CI: 1.08–1.21, *p* < 0.001) as the most significant independent predictors of MAFLD. These findings emphasize the strong contribution of obesity, poor glycemic control, and dyslipidemia to MAFLD development in T2DM patients, and highlight that the severity of MAFLD is multifactorial, due to metabolic, hepatic, and systemic factors. The identification of these predictors provides an important clue for risk stratification and management strategy based on disease severity.

### Age- and Sex-Stratified Prevalence of MAFLD

The prevalence of MAFLD was significantly higher in patients aged ≥ 60 years compared to those < 60 years (53.6% vs. 41.2%, *p* = 0.023), even after adjusting for BMI, HbA1c, and hypertension. This finding suggests that age is an independent risk factor for MAFLD, highlighting the need for targeted screening in older diabetic populations. In contrast, sex was not a significant predictor of MAFLD prevalence (*p* = 0.74), indicating that metabolic factors may play a more dominant role than sex-related differences in this cohort (Table 6).

**Table 6:** Age/sex stratification

Subgroup	MAFLD % (n/N)	Adjusted OR (95% CI)^a^	*p*-value
Age < 60	41.2% (72/175)	Ref	-
Age ≥ 60	53.6% (67/125)	1.65 (1.07–2.54)	0.023
Male	45.2% (62/137)	Ref	-
Female	47.1% (77/163)	1.08 (0.69–1.69)	0.74

^a^Adjusted for BMI, HbA1c, and hypertension

### Validation of MAFLD diagnosis: comparison of ultrasound and MRI-PDFF

Magnetic resonance imaging proton density fat fraction (MRI-PDFF) validation revealed that ultrasound (US) missed hepatic steatosis in 18% of cases, all of which had fat content below 15% (Table 7). Based on the observed sensitivity (84%) and correction factors from previous Egyptian studies, the estimated true prevalence of metabolic-associated fatty liver disease (MAFLD) in this cohort is 52.4% (95% confidence interval [CI]: 46–58%). This aligns with prior regional MRI-based prevalence estimates and highlights the limitations of US in detecting mild hepatic steatosis.

**Table 7:** Ultrasound vs. MRI-PDFF Discordance (*n* = 30)

Ultrasound	MRI-PDFF + (Steatosis Present)	MRI-PDFF − (No Steatosis)	Sensitivity	Estimated True MAFLD Prevalence
MAFLD +	22	3	84%	**52.4% (95% CI: 46–58%)**
MAFLD −	5	0	-	-

### Component analysis of NAFLD fibrosis score (NFS) in MAFLD risk prediction

Component analysis of the NAFLD Fibrosis Score (NFS) revealed that metabolic factors, BMI, and diabetes accounted for 72% of its variance, whereas fibrosis-specific markers, such as albumin, contributed only 14% (Table 8). This suggests that NFS primarily reflects metabolic dysfunction rather than true fibrosis severity. These findings support its reclassification as a ‘MAFLD risk score’ rather than a pure fibrosis marker in this cohort.

**Table 8:** NFS component contributions

Component	β-coefficient	*p*-value	% Variance Explained
BMI	+ 0.12	< 0.001	31%
Diabetes	+ 1.18	< 0.001	41%
Albumin	-0.64	< 0.001	14%

## Discussion

Diagnosing Metabolic-Associated Fatty Liver Disease (MAFLD), which is poorly understood by doctors and patients, is critical due to its potential progression to advanced liver diseases, including cirrhosis and hepatocellular carcinoma, as well as extrahepatic manifestations; these conditions are prevalent if they are linked to diabetes mellitus or obesity [30]. The primary strategy for the diagnosis of fatty liver is the detection of steatosis, usually by imaging studies. Ultrasound is generally the first choice because of its easy availability, but it has significant drawbacks. Among these are a low sensitivity of less than 20%, variability among observers, and difficulty differentiating steatosis from steatohepatitis or fibrosis [31]. A variety of diagnostic modalities, from histological to non-invasive methods, are available to identify and stratify MAFLD, each with its strengths and limitations. Generally, liver biopsy is considered the gold standard for diagnosis, since it provides an accurate histological grading of steatosis, inflammation, and fibrosis. However, its invasive nature, besides the risks of bleeding and infection, as well as variability in sampling, makes its routine use limited to patients with indeterminate or severe diseases who require confirmation. Additionally, the costs and logistical challenges associated with biopsies make them impractical for large-scale screening or routine monitoring [32].

Advanced imaging techniques consist of transient elastography (FibroScan) and magnetic resonance imaging-derived proton density fat fraction (MRI-PDFF) or elastography, which represent widely available, noninvasive, and reliable means to assess liver fat and fibrosis. They have a high degree of accuracy for the detection of early alterations in the structure and function of the liver. However, high costs, limited availability, and specialized equipment and expertise are the major drawbacks to their widespread use [32–34]. Emerging technologies, like bioelectrical impedance analysis tools (e.g., InBody), have shown promise but require further clinical validation [35]. Thus, the FIB-4 index, NAFLD Fibrosis Score (NFS), Hepatic Steatosis Index (HSI), Fatty Liver Index (FLI), and others have emerged as easy and non-invasive indices as an alternative for MAFLD diagnosis, screening, and monitoring. These scoring systems utilize easily accessible data, both clinically and in the laboratory, such as age, BMI, liver enzymes, and lipid profiles, to estimate fibrosis or steatosis probability [36]. The FIB-4 index and NFS have demonstrated great performance in stratifying risk for fibrosis, with well-defined thresholds categorizing the risks into low, indeterminate, or high risk of having advanced fibrosis [24, 26]. Considering the assessment of steatosis, HSI and FLI effectively monitored the accumulation of hepatic fat among populations with metabolic risks [37, 38]. These indices are inexpensive, widely available, and non-invasive, making them ideal for large-scale screening and longitudinal follow-up, especially in high-risk populations such as those with Type 2 Diabetes Mellitus [36].

Considering traditional predictors such as BMI, waist circumference, and dyslipidemia, a stepwise screening approach could enhance the efficiency of MAFLD detection. Using non-invasive indices like HSI and FLI as initial screening tools for hepatic steatosis, followed by NFS and FIB-4 for fibrosis risk stratification, could optimize clinical decision-making. This approach may reduce unnecessary imaging and improve resource utilization, particularly in resource-limited settings.

The current study underlines the importance of non-invasive indices, including FIB-4, NFS, HSI, and FLI, along with abdominal ultrasound, in the diagnosis and stratification of MAFLD in patients with T2DM. In the present study, MAFLD was diagnosed in the presence of hepatic steatosis detected by ultrasound imaging along with metabolic risk factors. Early identification of steatosis severity degree through non-invasive imaging like ultrasound could therefore serve as a critical tool in tailoring patient management, possibly guiding therapeutic decisions such as the initiation of pharmacologic interventions or more frequent monitoring. The four non-invasive scores (FIB-4, NFS, HSI, FLI) complement ultrasound by stratifying the risk of fibrosis or steatosis and supporting the diagnosis. These indices are critical for distinguishing the severity of liver involvement and for assessing liver health without invasive procedures, especially in resource-poor countries.

Our study included 300 patients with T2DM, and MAFLD was confirmed in 46.33% of participants (139/300) based on the presence of hepatic steatosis on ultrasound and metabolic dysfunction criteria, including T2DM and obesity. In the demographic analysis, it is seen that there was no significant difference between groups according to age, sex, smoking, or hypertension, while BMI and waist circumference were significantly higher in the MAFLD group. That means metabolic factors like obesity and visceral fat are highly important in MAFLD development compared to classic cardiovascular risk factors such as hypertension. Furthermore, the relationship between MAFLD and T2DM aligns with evidence suggesting that MAFLD itself exacerbates the risk of metabolic complications, including poor glycemic control and diabetes progression, as highlighted by a recent study emphasizing the distinct clinical implications of MAFLD compared to NAFLD​ ( 39).

This comprehensive analysis reports a global prevalence of type 2 diabetes among patients with MAFLD at approximately 28.3% [40]. The present study focused on an Egyptian cohort and found a MAFLD prevalence of 46.33% among T2DM patients, indicating a higher regional burden. This discrepancy underscores the significant impact of metabolic disorders in Egypt, likely due to factors such as high obesity rates and genetic predispositions. Unfortunately, MAFLD is a major public health concern in Egypt that affects more than 45% of the population [41]. A study conducted in the Fayoum Governorate found that the prevalence of MAFLD was 43.6%, indicating that it is widely distributed throughout the region [42]. The prevalence of diabetes among Egyptians is almost 70%, suggesting a close connection between MAFLD and metabolic diseases [41]. Furthermore, Egypt's high rates of obesity and diabetes contribute considerably to the Middle East and North Africa region's one of the highest global MAFLD prevalence rates, which is expected to reach 37% [43, 44]. This rise is closely linked to increasing obesity and type 2 diabetes mellitus (T2DM), as Egypt ranks among the top 10 countries with the highest obesity rates; 71.2% of adult men are overweight (26.4% obese), while 79.4% of adult women are overweight (48.4% obese). MAFLD progression often leads to severe complications, including cirrhosis and hepatocellular carcinoma (HCC), with MAFLD-related HCC cases in Egypt rising from 4.3% in 2010 to 20.6% in 2020 [41].

Overweight and obesity, expressed as increased values of BMI and waist circumference, are the leading risk factors for the development of MAFLD most prevailing chronic liver disease nowadays [45]. To date, dysfunctional visceral adipose tissue has been viewed as a crucial player in the pathogenesis of MAFLD. In the absence of the accumulation of visceral fat, it is very seldom that MAFLD occurs and may represent another condition [46]. The observed relation of higher BMI and waist circumference with the prevalence of MAFLD, as seen in our study, is in concert with global evidence linking adiposity with fatty liver disease. Even modest increases in BMI have been demonstrated to increase the risk of MAFLD [47], while the role of visceral adiposity in driving the continuum from obesity to MAFLD and metabolic dysfunction has also been emphasized [48]. The visceral fat plays an especially crucial role since obese MAFLD patients with T2DM reveal far more serious metabolic disturbances than their non-obese peers [49]. Diabetic MAFLD is also associated with a significant risk of hepatocellular carcinoma and mortality, necessitating the urgency for early screening in high-risk groups [50].

The importance of BMI and waist circumference collectively as critical predictors of MAFLD, and thereby a targeted intervention to mitigate obesity and metabolic dysfunction, cannot be overemphasized, especially in populations experiencing high rates of obesity and diabetes, like Egypt. Egyptian studies have consistently highlighted a high prevalence of MAFLD due to the dual burden of diabetes and obesity in the population. Their study also emphasized the critical role of BMI and visceral adiposity in MAFLD pathogenesis, further supporting our results. Additionally, they highlighted that genetic predisposition, such as variants in the PNPLA3 gene, might contribute to the higher prevalence of MAFLD in Egyptian patients [51, 52]. The Egyptian Clinical Practice Guidelines recommend screening for MAFLD in at-risk populations, particularly those with overweight/obesity, T2DM, or metabolic dysfunction [41].

Our findings indicate that age is an independent predictor of MAFLD prevalence, with significantly higher rates observed in patients aged ≥ 60 years. This aligns with previous studies suggesting that aging-related metabolic changes, including increased visceral adiposity and altered hepatic lipid metabolism, contribute to MAFLD pathogenesis [51, 52]. Interestingly, sex did not significantly influence MAFLD prevalence in this cohort, reinforcing the notion that metabolic risk factors such as BMI and glycemic control may play a more dominant role than sex-related hormonal differences in this population.

In our study, no significant relationships were present between hypertension and smoking. The lack of a significant difference in hypertension between groups in our study contrasts with previous findings, where hypertension was identified as a contributing factor to MAFLD [53]. This discrepancy may reflect regional variations in patient profiles or the unique characteristics of the Egyptian population. Several previous studies investigated the connection between smoking and MAFLD, with varying degrees of success. According to a study, smoking did not raise liver enzymes or cause MAFLD in those without chronic liver disease. Despite smoking's established link to metabolic disorders such as insulin resistance and diabetes mellitus, the researchers concluded that smoking had no direct impact on the occurrence of MAFLD [54]. However, other research showed that smoking is associated with an increased risk and progression of MAFLD, exacerbating liver damage, particularly in individuals with metabolic disorders [55–58]. Considering these contradictory results, more investigation is required to elucidate the connection between smoking and MAFLD. Meanwhile, controlling MAFLD still requires a focus on well-established risk factors, including obesity and glycemic management, particularly in populations where these illnesses are highly prevalent, rather than focusing solely on traditional cardiovascular risk factors.

In our analysis, hypertension and dyslipidemia were not independent predictors of MAFLD after adjusting for BMI and glycemic control. This finding is consistent with previous studies indicating that their influence on MAFLD is often mediated through broader metabolic dysfunction rather than being direct causal factors. Given the strong interrelation between metabolic syndrome components, further research is needed to determine whether hypertension and dyslipidemia contribute independently to MAFLD progression or serve as secondary markers of metabolic impairment.

Our study highlights the complex relationship between MAFLD and hematological and certain biochemical parameters in T2DM patients. The study indicated that total leukocyte count and platelet counts initially rise due to inflammation associated with early disease stages, but they are expected to decline as liver fibrosis advances. The results also showed a state of dyslipidemia, characterized by elevated LDL and TG and decreased HDL, which is closely linked to metabolic dysfunction in MAFLD, in addition to elevated liver enzymes (ALT, AST, and GGT), indicating hepatocellular injury. High HbA1c levels further underscore the role of poor glycemic control in MAFLD progression. The study findings also highlighted that decreased albumin levels signal early liver dysfunction.

In patients with metabolic-associated fatty liver disease (MAFLD), platelet count may differ throughout different stages of the disease. In the early stages, it could be within the normal range or slightly higher due to an increase in inflammatory activity, as platelets become activated and release pro-inflammatory mediators, contributing to hepatic inflammation. Platelet-leukocyte interactions are also activated, enhancing inflammatory responses in the liver. This interaction further contributes to the initial rise in platelet counts observed in MAFLD patients [59, 60]. With the advancement of liver fibrosis, there is a reduction in thrombopoietin-producing capability by the liver, reducing platelet production. Portal hypertension, which is the frequent result of advanced liver disease, leads to splenomegaly, and hence, the platelets will be sequestered in the enlarged spleen and reduce their circulation. Moreover, chronic inflammation and metabolic dysfunction suppress the activity of bone marrow, further aggravating thrombocytopenia. In advanced stages, increased inflammatory and immune responses accelerate platelet destruction. This decrease in platelet count thus becomes a marker of disease progression with significant fibrosis or cirrhosis, and the need for early identification and management to prevent complications such as portal hypertension or liver failure [60, 61]. Platelet-activating factor (PAF) is a lipid mediator involved in inflammation and platelet aggregation may also contribute to initial increases in platelet counts due to enhanced platelet activation and aggregation [62]. In addition, early MAFLD is associated with chronic low-grade inflammation, which may result in a slight increase in TLC via the activation of immune responses due to hepatic fat accumulation, and the Release of pro-inflammatory cytokines such as TNF-α and IL-6, which stimulate leukocyte production [29, 63].

Dyslipidemia is considered a very important factor linked with the development of MAFLD. Abnormal lipid levels, such as increased triglycerides and low HDL cholesterol or high LDL cholesterol, in previous studies, have proved to be important parameters in the advancement of this disease. It seems that at the early stage, fat has accumulated in the liver because of an imbalance between accumulation and breakdown, leading to inflammation and oxidative stress, stimulating the development of fatty liver. Elevated triglyceride levels can lead to increased hepatic fat deposition, while low HDL cholesterol impairs the liver's ability to clear lipids, both contributing to NAFLD development [64]. The presence of dyslipidemia in MAFLD is indicative of deteriorating liver function and can lead to more advanced stages of the disease, such as liver fibrosis. Therefore, understanding the relationship between dyslipidemia and MAFLD is essential for early diagnosis and effective management to improve patient outcomes [65].

In general, patients with MAFLD have high levels of liver enzymes, especially ALT, AST, and GGT, due to fatty degeneration and inflammation of the hepatocytes. Several studies are related to such levels and the severity of liver damage; hence, such biomarkers could easily become part of the monitoring of the course of the disease [66–68]. Liver enzymes were related to the severity of MAFLD and hence could be useful for follow-ups during treatment and assessing the disease. It is essential to use liver enzymes as an indication clinically in the early detection and management of MAFLD [68]. In MAFLD, cellular damage refers to hepatocyte injury due to fat accumulation, oxidative stress, and inflammation, promoting liver cell death and contributing to fibrosis. Cholestatic damage involves impairment in bile flow, where bile is accumulated in the liver; this is usually a feature of more advanced disease stages. Accordingly, in MAFLD, these mechanisms can promote the increase of liver injuries from simple to NASH and even further to cirrhosis; this is characterized by rises in liver enzymes (AST, ALT, GGT) that function as markers of the injury [69]. Albumin, a protein synthesized by the liver, is often low in patients with MAFLD and thus indicates early liver dysfunction, indicating impaired liver synthetic function [70]. Indeed, several studies have indicated that low albumin levels are associated with more advanced stages of MAFLD, including NASH and cirrhosis. Monitoring albumin can thus serve as an early marker for liver injury and a predictor of disease severity in MAFLD patients [71, 72].

Previous studies stated that HbA1c may be presented as a potential biomarker for MAFLD presence and severity in examination with other anthropometric measures in the adult population, owing to the positive correlation between HbA1c and the development of MAFLD, suggesting that poor glycemic control is contributing to the progression toward liver disease. High levels of HbA1c indicate sustained hyperglycemia and insulin resistance, which are considered central factors in the pathogenesis of MAFLD [73, 74]. These findings were supported by results from research that showed the following: higher HbA1c is associated with increased accumulation of liver fat and inflammation in metabolic disorders [75].

Our study found no significant differences in creatinine or urea levels between MAFLD and non-MAFLD patients, suggesting that kidney function was preserved in the study cohort. Previous studies indicated that kidney function remains largely unaffected in the early stages of MAFLD, as measured by creatinine and urea levels. However, as the disease progresses, particularly with the onset of fibrosis or cirrhosis, renal function may deteriorate due to systemic factors such as increased inflammation, metabolic disturbances, and hypertension, highlighting the emerging MAFLD-Renal Syndrome. Therefore, while kidney function appears stable initially, long-term monitoring is essential to detect early signs of kidney involvement, which can significantly impact patient outcomes as MAFLD advances [76, 77].

In this study, the prevalence of MAFLD was 46.33% among participants with Type 2 Diabetes Mellitus (T2DM), the distribution of hepatic steatosis severity revealed that most patients had moderate steatosis (49.64%) as manifested by a moderate increase of liver echogenicity with a slightly impaired appearance of the portal vein wall and the diaphragm, followed by mild steatosis (30.94%) as manifested by a slight and diffuse increase of liver echogenicity with normal visualization of the diaphragm and the portal vein wall, and severe steatosis (19.42%) as manifested by marked increase of liver echogenicity with poor or no visualization of the portal vein wall, diaphragm, and posterior part of the right liver lobe [21]. This stratification is clinically significant as it helps prioritize patients for more intensive management. Moderate and severe steatosis is often associated with higher risks of fibrosis progression, metabolic complications, and poorer long-term outcomes, warranting closer monitoring and intervention.

While conventional ultrasound is available everywhere and relatively inexpensive for detecting steatosis and grading its severity, it is insensitive to detect mild steatosis (< 20% fat content), does not allow the quantification of the fat content with enough accuracy to differentiate steatosis from fibrosis or steatohepatitis, and its operator dependency [78]. Thus, advanced modalities such as quantitative ultrasound (QUS), magnetic resonance imaging-derived proton density fat fraction (MRI-PDFF), and transient elastography allow for more precise quantification of liver fat and fibrosis based on objective assessment of the liver fat via parameters like attenuation and backscatter coefficients [79, 80]. These techniques, unfortunately, become less accessible due to the cost and availability that limit their extensive application [81], especially in resource-limited settings like Egypt, where ultrasound remains a mainstay for the diagnosis of MAFLD.

A validation substudy using MRI-PDFF (*n* = 30) indicated that ultrasound failed to detect mild steatosis in 18% of cases, primarily in patients with lower hepatic fat content. Consequently, the true prevalence of MAFLD in this cohort may be underestimated. Given ultrasound’s limited sensitivity for early steatosis, its use as a sole screening tool should be interpreted with caution. Integrating additional non-invasive modalities, such as controlled attenuation parameter (CAP) via FibroScan or MRI-PDFF, may improve detection accuracy, particularly in patients at high metabolic risk.

Our findings align with EASL’s recommendation for systematic screening of high-risk T2DM patients, contrasting with AASLD’s more selective approach. Given Egypt’s high prevalence of both T2DM (17.2%) and MAFLD (~ 46%), early detection through non-invasive indices is a feasible, low-cost strategy to optimize resource allocation and prevent disease progression. Given the high burden of MAFLD in Egypt, integrating non-invasive indices into routine diabetes screening protocols could improve early detection. Current guidelines differ in their recommendations for MAFLD screening in T2DM patients. The European Association for the Study of the Liver (EASL) advocates screening in high-risk individuals, while the American Association for the Study of Liver Diseases (AASLD) suggests a case-by-case approach. Our findings support a proactive screening strategy in line with EASL recommendations, especially in resource-limited settings where cost-effective, non-invasive screening tools can improve early identification and timely intervention.

Our validation substudy using MRI-PDFF confirmed that ultrasound underestimates the prevalence of MAFLD, missing 18% of cases with mild steatosis. When adjusting for ultrasound sensitivity (84%), the estimated true prevalence of MAFLD in our cohort was 52.4% (95% CI: 46–58%). This aligns with regional studies using MRI-based assessment. Given ultrasound’s limitations, particularly in detecting mild hepatic steatosis, integrating additional non-invasive biomarkers or more sensitive imaging techniques, such as transient elastography, may improve diagnostic accuracy. Future studies should explore the cost-effectiveness of incorporating these methods in routine MAFLD screening among high-risk populations.

In this context, non-invasive indices, including the FIB-4 index, NAFLD Fibrosis Score (NFS), Hepatic Steatosis Index (HSI), and Fatty Liver Index (FLI), are gaining popularity as practical alternatives. These indices, coupled with ultrasound findings, are used to improve diagnostic and management accuracy and to provide reliable insights into the presence and severity of liver damage, especially among populations with limited resources because such indices utilize readily available clinical and laboratory data, such as age, BMI, liver enzymes, and lipid profiles, to provide reliable insights into the presence and severity of liver damage [24, 26, 36].

Our study explained the role of such non-invasive indices in liver health assessment and stratification of patients with T2DM based on fibrosis and steatosis risk. Among MAFLD patients, FIB-4, NFS, HSI, and FLI levels were significantly higher compared to their non-MAFLD counterparts, indicating that these could distinguish between the groups. The diagnostic performance, tested by sensitivity, specificity, and ROC analysis, was as follows: NFS had the best accuracy in detecting fibrosis with an AUC of 0.964, while for steatosis assessment, HSI and FLI showed comparable performance with AUCs of 0.847 and 0.835, respectively. These indices also showed strong sensitivity and moderate specificity at the optimal cutoff values, where FIB-4 had the highest sensitivity for detecting fibrosis at 83%, while HSI demonstrated 80% sensitivity for assessing steatosis.

The diagnostic performance of non-invasive indices observed in our study aligns with findings in existing literature. For fibrosis detection, a study identified FIB-4 and NFS diagnostic performance concerning liver fibrosis in NAFLD. The FIB-4 score represented 82% sensitivity and 76% specificity, with an AUC of 0.85, indicating that it was reliable for distinguishing the presence of advanced fibrosis. For its part, NFS showed a sensitivity of 84% and a specificity of 79%, with an AUC of 0.88. Both tools performed well in this regard, as supported by the ROC analysis showing high accuracy for the prediction of fibrosis [82]. A meta-analysis of 36 studies on biopsy-proven NAFLD involving 14,992 patients found that the FIB-4 score had a sensitivity of 69%, specificity of 64%, and an AUC of 0.76 for predicting ≥ F3 fibrosis. For NFS, sensitivity was 70%, specificity 61%, with an AUC of 0.74 for predicting ≥ F3 fibrosis. The positive likelihood ratios (LR +) for FIB-4 and NFS were 1.96 and 1.83, respectively, while their negative likelihood ratios (LR–) were 0.47 and 0.48 [83]. In a study on noninvasive diagnostic indices for MAFLD, the Hepatic Steatosis Index (HSI) and Fatty Liver Index (FLI) were evaluated. The AUROC for HSI was 0.874 (95% CI: 0.865–0.883), while FLI showed an AUROC of 0.884 (95% CI: 0.876–0.89. HSI's specificity at a high cut-off (> 36) was 94.4%, with a sensitivity of 93.4% at a low cut-off (< 30). FLI demonstrated a specificity of 98.4% at a high cut-off (> 60), but a lower sensitivity of 68.8% at a low cut-off (< 30). Both indices displayed strong diagnostic potential for MAFLD detection [84].

Given that ultrasound served as the reference standard, our ROC analysis reflects the ability of non-invasive indices to approximate ultrasound-based MAFLD detection rather than biopsy-confirmed liver pathology. The reliance on ultrasound may result in underestimation of mild steatosis cases, reinforcing the need for future validation against more sensitive imaging techniques such as MRI-PDFF or transient elastography.

In clinical practice, these cutoffs serve as screening tools where patients exceeding them may require further evaluation. Individuals with HSI ≥ 36 or FLI ≥ 60 may benefit from imaging confirmation to assess steatosis severity [27, 28], while those with NFS > 0.675 or FIB-4 > 1.45 should be considered at risk for fibrosis and may require hepatology referral [24, 26]. For patients falling into indeterminate risk categories, combining multiple indices or incorporating additional imaging modalities can enhance risk stratification and guide clinical decision-making.

In our study, the FIB-4 and NFS scores, which assess liver fibrosis, showed intermediate risk categories for both. Specifically, the FIB-4 score has a mean of 1.94 ± 0.81, placing patients in the intermediate risk category (1.45–3.25), and the NFS score has a mean of 0.56 ± 1.24, placing patients in the indeterminate risk category (-1.455 to 0.675), suggesting that while there is evidence of potential fibrosis, it is not definitive. Meanwhile, the HSI and FLI, which assess liver steatosis, indicated a high probability of MAFLD. The HSI score has a mean of 38.31 ± 6.93, placing patients in the high probability category (> 36), and the FLI score has a mean of 68.78 ± 29.98, placing patients in the high probability category (≥ 60), indicating a high probability of MAFLD. This discrepancy points to a situation where patients show significant liver steatosis, while the risk of fibrosis is not extensive, thus probably presenting an early stage of MAFLD. These findings were in concert with our ultrasonographic findings, where it was determined that most of the patients were in the mild and moderate stages of steatosis, 30.94% and 49.64%, respectively. Besides, it agrees with the laboratory outcome where the kidney function remained stable, the platelet count was higher, and the white blood cells rose to a high normal limit, with low levels of albumin. All these establish the fact that the patients are at an early stage of MAFLD. Thus, the combination of laboratory tests, ultrasound, and the four diagnostic scores supports and strengthens each other for more accurate confirmation results.

Our findings indicate that NFS primarily reflects metabolic dysfunction rather than independently confirmed fibrosis. A component analysis showed that BMI and diabetes contributed 72% of the variance in NFS, whereas fibrosis-specific markers such as albumin contributed only 14%. Given this, we propose reclassifying NFS as a ‘MAFLD risk score’ in T2DM rather than a direct fibrosis marker. Scores above 0.675 in this cohort likely indicate a high probability of metabolic liver disease rather than true advanced fibrosis. Future studies should evaluate modified fibrosis index cutoffs specific to MAFLD patients to improve diagnostic precision.

In our study, given the high probability of MAFLD in the studied population, it was crucial to investigate the predictors associated with patients with T2DM for the early detection and prevention of complications. Several predictors were identified in our study for the presence and severity of MAFLD in patients with type 2 diabetes. The main factors influencing the presence of MAFLD were obesity (increased BMI), visceral adiposity (increased waist circumference), poor glycemic control (increased HbA1c), increased liver enzymes (ALT, AST, GGT), lower albumin levels, and dyslipidemia (TG, LDL, HDL); all of them had statistically significant odds ratios for the diagnosis of MAFLD. For severity, predictors, especially BMI, HbA1c, and liver enzymes, were important. Comparing the severities predictors between Grade 2 vs. Grade 1 and Grade 3 vs. Grade 1, suggested that some biomarkers, including BMI, HbA1c, liver enzymes, and lipid profiles, are associated with disease progression from Grade 1 to Grade 2 and Grade 3, and proved that such factors are significant predictors of the severity of MAFLD even in its early stage.

The strong association of BMI, HbA1c, and triglycerides with MAFLD highlights the importance of metabolic control in disease prevention. Since these are modifiable risk factors, interventions targeting weight loss, glycemic control, and lipid management may significantly reduce MAFLD risk in T2DM patients. These findings emphasize the need for personalized treatment strategies that prioritize metabolic optimization to prevent disease progression [85].

Previous studies identified several predictors of metabolic-associated fatty liver disease (MAFLD) in adults, including increased body mass index (BMI), waist circumference (WC), and higher serum levels of triglycerides (TG), total cholesterol (TC), and alanine aminotransferase (AST). These factors were found to significantly increase the likelihood of MAFLD, with odds ratios indicating strong associations [85–87].

The presence and severity predictors are crucial for early detection and stratification of MAFLD, allowing targeted interventions aimed at preventing complications such as cirrhosis and liver failure. Non-invasive scores, including FIB-4, NFS, HSI, and FLI, provide pragmatic tools for screening, enabling timely and individualized management of high-risk subjects, especially those with T2DM. Long-term outcomes can be improved by regular monitoring of these scores and associated biomarkers, guiding effective interventions for obesity, dyslipidemia, and glycemic control.

Recent studies highlight the role of gut microbiota in MAFLD pathogenesis via the gut-liver axis. Dysbiosis has been linked to increased intestinal permeability, endotoxemia, and systemic inflammation, all of which contribute to hepatic fat accumulation and insulin resistance. Microbiota-targeted interventions, including probiotics and prebiotics, have demonstrated potential in modifying disease progression [88]. Future studies should explore microbiome-based therapeutic strategies for MAFLD prevention and treatment, particularly in resource-limited settings. Probiotic supplementation has been investigated as a potential adjunctive therapy for MAFLD, with studies demonstrating improvements in hepatic fat content and metabolic markers. Given the accessibility and cost-effectiveness of probiotics, they may serve as a practical intervention in resource-limited settings [89]. Future research should explore the long-term efficacy and safety of probiotic-based interventions as part of comprehensive MAFLD management strategies.

This study provides novel insights into the screening and diagnostic performance of non-invasive indices for MAFLD in patients with type 2 diabetes mellitus (T2DM) within an Egyptian population. To our knowledge, this is the first study in Egypt to evaluate all four widely used non-invasive indices—HSI, FLI, FIB-4, and NFS—in parallel, allowing for a comprehensive assessment of their utility in clinical practice. Our findings offer population-specific data on MAFLD prevalence and highlight the practicality of integrating these indices into routine screening protocols. Given the high burden of T2DM and MAFLD in Egypt, this study supports a cost-effective, scalable approach to early detection, aligning with global recommendations for systematic MAFLD screening in high-risk populations.

This study has several limitations that should be acknowledged. First, the cross-sectional design limits the ability to establish causal relationships between metabolic risk factors and MAFLD severity. Longitudinal studies are needed to assess disease progression and the impact of interventions over time. Second, the study was conducted at a single tertiary diabetes center, which may restrict generalizability. However, patient characteristics closely align with national registry data, supporting external validity. A larger multi-center study would enhance the reliability and broader applicability of the findings. Third, the study relied on non-invasive indices and ultrasound for diagnosing MAFLD and assessing fibrosis risk. While these tools are widely used, ultrasound has limited sensitivity for detecting mild hepatic steatosis, and fibrosis scores such as FIB-4 and NFS were not validated against liver biopsy, which may have led to underestimation of MAFLD prevalence and fibrosis misclassification. Additionally, ultrasound is operator-dependent, introducing variability in diagnosis. To mitigate this, all scans were conducted by a single radiologist, but future studies should incorporate standardized imaging protocols, transient elastography, or liver biopsy for improved diagnostic accuracy. Finally, unmeasured confounders such as medication use, dietary habits, and physical activity were not accounted for, despite their potential impact on hepatic fat accumulation and fibrosis risk. Future research should integrate histopathological validation, advanced imaging techniques, and longitudinal follow-up to improve diagnostic precision and better understand disease progression.

Alcohol consumption is a known factor that can exacerbate metabolic dysfunction and liver injury in patients with T2DM. However, in Egypt, alcohol consumption is relatively uncommon due to cultural and religious factors, which limit its impact on MAFLD prevalence in this population. While alcohol intake data were not collected in this study, future research in populations with higher alcohol consumption could further elucidate its role in MAFLD progression.

## Conclusion

Our study emphasized the importance of non-invasive diagnostic scores and clinical parameters for evaluating the presence and severity of MAFLD in individuals with Type 2 diabetes. Early detection and stratification through tools like ultrasound and non-invasive scores such as FIB-4, NFS, HSI, and FLI, alongside clinical markers, is essential for the early management of patients at risk, providing timely interventions to prevent disease progression and improve long-term outcomes. Our findings support a stepwise screening approach for MAFLD in T2DM patients: (1) HSI ≥ 36 or FLI ≥ 60 suggests high steatosis probability, warranting lifestyle intervention and potential imaging confirmation; (2) NFS > 0.675 or FIB-4 > 1.45 indicates a higher fibrosis risk, suggesting the need for hepatology referral. In regions with a high burden of T2DM, integrating non-invasive indices into routine diabetic care aligns with EASL recommendations and offers a cost-effective, scalable approach for early MAFLD detection, particularly in resource-limited settings.

## Data Availability

All relevant data are included in this published article.

## References

[1] Boccatonda A, Andreetto L, D’Ardes D, Cocco G, Rossi I, Vicari S, Schiavone C, Cipollone F, Guagnano MT. From NAFLD to MAFLD: definition, pathophysiological basis, and cardiovascular implications. Biomedicines. 2023;11(3):883. 36979861 10.3390/biomedicines11030883 PMC10046146

[2] Eslam M, Newsome PN, Sarin SK, Anstee QM, Targher G, Romero-Gomez M, Zelber-Sagi S, Wong VW, Dufour JF, Schattenberg JM, Kawaguchi T. A new definition for metabolic dysfunction-associated fatty liver disease: an international expert consensus statement. J Hepatol. 2020;73(1):202–9. 32278004 10.1016/j.jhep.2020.03.039

[3] Miao L, Targher G, Byrne CD, Cao YY, Zheng MH. Current status and future trends of the global burden of MASLD. Trends Endocrinol Metab. 2024;35(8):697–707. 38429161 10.1016/j.tem.2024.02.007

[4] Clemente-Suárez VJ, Martín-Rodríguez A, Redondo-Flórez L, López-Mora C, Yáñez-Sepúlveda R, Tornero-Aguilera JF. New insights and potential therapeutic interventions in metabolic diseases. Int J Mol Sci. 2023;24(13):10672. 37445852 10.3390/ijms241310672 PMC10342188

[5] Wang D, Xu Y, Zhu Z, Li Y, Li X, Li Y, Shen H, Wu W, Liu Y, Han C. Changes in the global, regional, and national burdens of NAFLD from 1990 to 2019: a systematic analysis of the global burden of disease study 2019. Front Nutr. 2022;9:1047129. 36618688 10.3389/fnut.2022.1047129 PMC9811393

[6] Hagström H, Vessby J, Ekstedt M, Shang Y. 99% of patients with NAFLD meet MASLD criteria, and natural history is therefore identical. J Hepatol. 2024;80(2):e76–7. 37678723 10.1016/j.jhep.2023.08.026

[7] Zhou XD, Targher G, Byrne CD, Somers V, Kim SU, Chahal CA, Wong VW, Cai J, Shapiro MD, Eslam M, Steg PG. An international multidisciplinary consensus statement on MAFLD and the risk of CVD. Hep Intl. 2023;17(4):773–91. 10.1007/s12072-023-10543-8 PMC10198034 37204656

[8] Kaya E, Yilmaz Y. Metabolic-associated fatty liver disease (MAFLD): a multi-systemic disease beyond the liver. J Clin Transl Hepatol. 2021;10(2):329. 35528971 10.14218/JCTH.2021.00178 PMC9039705

[9] Huang DQ, Terrault NA, Tacke F, Gluud LL, Arrese M, Bugianesi E, Loomba R. Global epidemiology of cirrhosis—etiology, trends, and predictions. Nat Rev Gastroenterol Hepatol. 2023;20(6):388–98. 36977794 10.1038/s41575-023-00759-2 PMC10043867

[10] Vetrano E, Rinaldi L, Mormone A, Giorgione C, Galiero R, Caturano A, Nevola R, Marfella R, Sasso FC. Non-alcoholic fatty liver disease (NAFLD), type 2 diabetes, and non-viral hepatocarcinoma: pathophysiological mechanisms and new therapeutic strategies. Biomedicines. 2023;11(2):468. 36831004 10.3390/biomedicines11020468 PMC9953066

[11] Kamada Y, Munekage K, Nakahara T, Fujii H, Sawai Y, Doi Y, Hyogo H, Sumida Y, Imai Y, Miyoshi E, Ono M. The FIB-4 index predicts the development of liver-related events, extrahepatic cancers, and coronary vascular disease in patients with NAFLD. Nutrients. 2022;15(1):66. 36615725 10.3390/nu15010066 PMC9824239

[12] Gotlieb N, Schwartz N, Zelber-Sagi S, Chodick G, Shalev V, Shibolet O. Longitudinal decrease in platelet counts as a surrogate marker of liver fibrosis. World J Gastroenterol. 2020;26(38):5849. 33132639 10.3748/wjg.v26.i38.5849 PMC7579756

[13] Amernia B, Moosavy SH, Banookh F, Zoghi G. FIB-4, APRI, and AST/ALT ratio compared to FibroScan for the assessment of hepatic fibrosis in patients with non-alcoholic fatty liver disease in Bandar Abbas. Iran BMC Gastroenterology. 2021;21:1–7. 10.1186/s12876-021-02038-3 PMC8642865 34861841

[14] O’Hara G, Mokaya J, Hau JP, Downs LO, McNaughton AL, Karabarinde A, Asiki G, Seeley J, Matthews PC, Newton R. Liver function tests and fibrosis scores in a rural population in Africa: a cross-sectional study to estimate the burden of disease and associated risk factors. BMJ Open. 2020;10(3):e032890. 32234740 10.1136/bmjopen-2019-032890 PMC7170602

[15] Petzold G. Role of ultrasound methods for the assessment of NAFLD. J Clin Med. 2022;11(15):4581. 35956196 10.3390/jcm11154581 PMC9369745

[16] Mumtaz S, Schomaker N, Von Roenn N. Pro: Noninvasive imaging has replaced biopsy as the gold standard in the evaluation of nonalcoholic fatty liver disease. Clinical Liver Disease. 2019;13(4):111–3. 31061704 10.1002/cld.750 PMC6491026

[17] Amini-Salehi E, Letafatkar N, Norouzi N, Joukar F, Habibi A, Javid M, Sattari N, Khorasani M, Farahmand A, Tavakoli S, Masoumzadeh B. Global prevalence of nonalcoholic fatty liver disease: an updated review Meta-analysis comprising a Population of 78 million from 38 countries. Arch Med Res. 2024;55(6):103043. 39094335 10.1016/j.arcmed.2024.103043

[18] Younossi ZM, Golabi P, de Avila L, Paik JM, Srishord M, Fukui N, Qiu Y, Burns L, Afendy A, Nader F. The global epidemiology of NAFLD and NASH in patients with type 2 diabetes: a systematic review and meta-analysis. J Hepatol. 2019;71(4):793–801. 31279902 10.1016/j.jhep.2019.06.021

[19] Dasarathy S, Dasarathy J, Khiyami A, Joseph R, Lopez R, McCullough AJ. Validity of real-time ultrasound in the diagnosis of hepatic steatosis: a prospective study. J Hepatol. 2009;51(6):1061–7. 19846234 10.1016/j.jhep.2009.09.001 PMC6136148

[20] Gaharwar R, Trikha S, Margekar SL, Jatav OP, Ganga PD. Study of clinical profile of patients of nonalcoholic fatty liver disease and its association with metabolic syndrome. J Assoc Physicians India. 2015;63(1):12–6. 26591121

[21] Ferraioli G, Monteiro LB. Ultrasound-based techniques for the diagnosis of liver steatosis. World J Gastroenterol. 2019;25(40):6053. 31686762 10.3748/wjg.v25.i40.6053 PMC6824276

[22] Wang D, Zhou BY, Xiang L, Chen XY, Feng JX. Alanine aminotransferase as a risk marker for new-onset metabolic dysfunction-associated fatty liver disease. World J Gastroenterol. 2024;30(25):3132. 39006380 10.3748/wjg.v30.i25.3132 PMC11238669

[23] Xuan Y, Wu D, Zhang Q, Yu Z, Yu J, Zhou D. Elevated ALT/AST ratio as a marker for NAFLD risk and severity: insights from a cross-sectional analysis in the United States. Front Endocrinol. 2024;26(15):1457598. 10.3389/fendo.2024.1457598 PMC11381241 39253584

[24] Sterling RK, Lissen E, Clumeck N, Sola R, Correa MC, Montaner J, Sulkowski MS, Torriani FJ, Dieterich DT, Thomas DL, Messinger D. Development of a simple noninvasive index to predict significant fibrosis in patients with HIV/HCV coinfection. Hepatology. 2006;43(6):1317–25. 16729309 10.1002/hep.21178

[25] Eletreby R, Abdellatif Z, Gaber Y, Ramadan A, Ahmad N, Khattab H, Said M, Saad Y. Validity of routine biochemical and ultrasound scores for prediction of hepatic fibrosis and steatosis in NAFLD. Egyptian Liver Journal. 2021;11:1.

[26] Angulo P, Hui JM, Marchesini G, Bugianesi E, George J, Farrell GC, Enders F, Saksena S, Burt AD, Bida JP, Lindor K. The NAFLD fibrosis score: a noninvasive system that identifies liver fibrosis in patients with NAFLD. Hepatology. 2007;45(4):846–54. 17393509 10.1002/hep.21496

[27] Lee JH, Kim D, Kim HJ, Lee CH, Yang JI, Kim W, Kim YJ, Yoon JH, Cho SH, Sung MW, Lee HS. Hepatic steatosis index: a simple screening tool reflecting nonalcoholic fatty liver disease. Dig Liver Dis. 2010;42(7):503–8. 19766548 10.1016/j.dld.2009.08.002

[28] Bedogni G, Bellentani S, Miglioli L, Masutti F, Passalacqua M, Castiglione A, Tiribelli C. The Fatty Liver Index: a simple and accurate predictor of hepatic steatosis in the general population. BMC Gastroenterol. 2006;6:1–7. 17081293 10.1186/1471-230X-6-33 PMC1636651

[29] Lee YJ, Lee HR, Shim JY, Moon BS, Lee JH, Kim JK. Relationship between white blood cell count and nonalcoholic fatty liver disease. Dig Liver Dis. 2010;42(12):888–94. 20472517 10.1016/j.dld.2010.04.005

[30] Fouad Y, Alboraie M, Shiha G. Epidemiology and diagnosis of metabolic dysfunction-associated fatty liver disease. Hep Intl. 2024;5:1–7. 10.1007/s12072-024-10704-3 PMC11450050 38967907

[31] Desai NK, Harney S, Raza R, Al-Ibraheemi A, Shillingford N, Mitchell PD, Jonas MM. Comparison of controlled attenuation parameter and liver biopsy to assess hepatic steatosis in pediatric patients. J Pediatr. 2016;1(173):160–4. 10.1016/j.jpeds.2016.03.021 PMC5105890 27039224

[32] Caussy C, Reeder SB, Sirlin CB, Loomba R. Noninvasive, quantitative assessment of liver fat by MRI-PDFF as an endpoint in NASH trials. Hepatology. 2018;68(2):763–72. 29356032 10.1002/hep.29797 PMC6054824

[33] El-Assaly H, El-Adawy LA, El-Latif RS, Fawzi MM, Osama A. Quantification of liver fat using non-invasive MRI-PDFF technique versus guided biopsy in potential liver donor. Egyptian J Radiol Nuclear Med. 2024;55(1):153.

[34] Hu R, Wu B, Wang C, Wu Z, Zhang X, Chen X, Lu G, Yuan K. Assessment of transient elastography in diagnosing MAFLD and the early effects of sleeve gastrectomy on MAFLD among the Chinese population. Int J Surg. 2024;110(4):2044–54. 38215263 10.1097/JS9.0000000000001078 PMC11020019

[35] Choi JW, Yoo JJ, Kim SG, Kim YS. Bioelectrical impedance analysis can be an effective tool for screening fatty liver in patients with suspected liver disease. Healthcare. 2022;10(11):2268. 36421592 10.3390/healthcare10112268 PMC9690130

[36] Abdelhameed F, Kite C, Lagojda L, Dallaway A, Chatha KK, Chaggar SS, Dalamaga M, Kassi E, Kyrou I, Randeva HS. Non-invasive scores and serum biomarkers for fatty liver in the era of metabolic dysfunction-associated steatotic liver disease (MASLD): a comprehensive review From NAFLD to MAFLD and MASLD. Curr Obes Rep. 2024;29:1–22. 10.1007/s13679-024-00574-z PMC11306269 38809396

[37] Zhou JH, Cai JJ, She ZG, Li HL. Noninvasive evaluation of nonalcoholic fatty liver disease: Current evidence and practice. World J Gastroenterol. 2019;25(11):1307. 30918425 10.3748/wjg.v25.i11.1307 PMC6429343

[38] Chen LW, Huang PR, Chien CH, Lin CL, Chien RN. A community-based study on the application of fatty liver index in screening subjects with nonalcoholic fatty liver disease. J Formos Med Assoc. 2020;119(1):173–81. 30981560 10.1016/j.jfma.2019.03.016

[39] Park SH, Park J, Kwon SY, Lee YB, Kim G, Hur KY, Koh J, Jee JH, Kim JH, Kang M, Jin SM. Increased risk of incident diabetes in patients with MAFLD not meeting the criteria for NAFLD. Sci Rep. 2023;13(1):10677. 37393407 10.1038/s41598-023-37858-8 PMC10314928

[40] Cao L, An Y, Liu H, Jiang J, Liu W, Zhou Y, Shi M, Dai W, Lv Y, Zhao Y, Lu Y. Global epidemiology of type 2 diabetes in patients with NAFLD or MAFLD: a systematic review and meta-analysis. BMC Med. 2024;22(1):101. 38448943 10.1186/s12916-024-03315-0 PMC10919055

[41] Fouad Y, Esmat G, Elwakil R, Zakaria S, Yosry A, Waked I, El-Razky M, Doss W, El-Serafy M, Mostafa E, Anees M. The Egyptian clinical practice guidelines for the diagnosis and management of metabolic-associated fatty liver disease. Saudi Journal of Gastroenterology. 2022;28(1):3–20. 35083973 10.4103/sjg.sjg_357_21 PMC8919931

[42] Fouad EG, Gomaa AA, Hassan EA, Tawfic M, Massoud M, Fouad Y, Fares E. Study of prevalence of Metabolic associated Fatty Liver Disease (MAFLD) in Fayoum Governorate. Labyrinth: Fayoum Journal of Science and Interdisciplinary Studies. 2024;2(1):42–7.

[43] Younossi ZM, Golabi P, Paik J, Owrangi S, Yilmaz Y, El-Kassas M, Alswat K, Alqahtani SA. Prevalence of metabolic dysfunction-associated steatotic liver disease in the Middle East and North Africa. Liver Int. 2024;44(4):1061–70. 38305642 10.1111/liv.15852

[44] Shalaby MF, Fouad YM, AbdelAzeem OA, Moneer MM, Higazi MM, Ahmed E, Saedi AA, Mahmoud SR, Mahmoud ES, Semeda NM. Prevalence and possible risk factors of metabolic associated fatty liver disease (MAFLD) in non-obese individuals in El-Minia Governorate-Egypt. Egypt J Hospital Med. 2023;92:5756–62.

[45] Gastaldelli A, Cusi K. From NASH to diabetes and from diabetes to NASH: mechanisms and treatment options. JHEP reports. 2019;1(4):312–28. 32039382 10.1016/j.jhepr.2019.07.002 PMC7001557

[46] Francque SM, Dirinck E. NAFLD prevalence and severity in overweight and obese populations. The Lancet Gastroenterology & Hepatology. 2023;8(1):2–3. 36400096 10.1016/S2468-1253(22)00375-2

[47] Liu M, Wang J, Zeng J, Cao X, He Y. Association of NAFLD with diabetes and the impact of BMI changes: a 5-year cohort study based on 18,507 elderly. J Clin Endocrinol Metab. 2017;102(4):1309–16. 28324002 10.1210/jc.2016-3440

[48] Godoy-Matos AF, Silva Júnior WS, Valerio CM. NAFLD as a continuum: from obesity to metabolic syndrome and diabetes. Diabetol Metab Syndr. 2020;12:1–20. 32684985 10.1186/s13098-020-00570-y PMC7359287

[49] Dang SW, Gao L, Li YJ, Zhang R, Xu J. Metabolic characteristics of non-obese and obese metabolic dysfunction-associated fatty liver disease in type 2 diabetes mellitus and its association with diabetic peripheral neuropathy and diabetic retinopathy. Front Med. 2023;10:1216412. 10.3389/fmed.2023.1216412 PMC10566373 37828942

[50] Kim MN, Han K, Yoo J, Hwang SG, Zhang X, Ahn SH. Diabetic MAFLD is associated with increased risk of hepatocellular carcinoma and mortality in chronic viral hepatitis patients. Int J Cancer. 2023;153(8):1448–58. 37439276 10.1002/ijc.34637

[51] El-Kassas M, Cabezas J, Coz PI, Zheng MH, Arab JP, Awad A. Nonalcoholic fatty liver disease: current global burden. Semin Liver Dis. 2022;42(03):401–12. Thieme Medical Publishers, Inc. 35617968 10.1055/a-1862-9088

[52] Tharwat M, Medhat MA, El-Kassas M. The NAFLD–MAFLD debate through the lens of the Arab world. Saudi J Gastroenterol. 2022;28(6):413–6. 36124490 10.4103/sjg.sjg_314_22 PMC9843511

[53] Wu Y, Hu H, Cai J, Chen R, Zuo X, Cheng H, Yan D. Association of hypertension and incident diabetes in Chinese adults: a retrospective cohort study using propensity-score matching. BMC Endocr Disord. 2021;21(1):87. 33926442 10.1186/s12902-021-00747-0 PMC8082672

[54] Chavez-Tapia NC, Lizardi-Cervera J, Perez-Bautista O, Ramos-Ostos MH, Uribe M. Smoking is not associated with nonalcoholic fatty liver disease. World J Gastroenterol: WJG. 2006;12(32):5196. 16937532 10.3748/wjg.v12.i32.5196 PMC4088019

[55] Okamoto M, Miyake T, Kitai K, Furukawa S, Yamamoto S, Senba H, Kanzaki S, Deguchi A, Koizumi M, Ishihara T, Miyaoka H. Cigarette smoking is a risk factor for the onset of fatty liver disease in nondrinkers: A longitudinal cohort study. PLoS ONE. 2018;13(4):e0195147. 29664906 10.1371/journal.pone.0195147 PMC5903610

[56] Akhavan Rezayat A, Dadgar Moghadam M, Ghasemi Nour M, Shirazinia M, Ghodsi H, Rouhbakhsh Zahmatkesh MR, Tavakolizadeh Noghabi M, Hoseini B, Akhavan Rezayat K. Association between smoking and non-alcoholic fatty liver disease: a systematic review and meta-analysis. SAGE open medicine. 2018;6:2050312117745223. 29399359 10.1177/2050312117745223 PMC5788091

[57] Jung HS, Chang Y, Kwon MJ, Sung E, Yun KE, Cho YK, Shin H, Ryu S. Smoking and the risk of non-alcoholic fatty liver disease: a cohort study. Am J Gastroenterol. 2019;114(3):453–63. 30353055 10.1038/s41395-018-0283-5

[58] Rutledge SM, Asgharpour A. Smoking and liver disease. Gastroenterol Hepatol. 2020;16(12):617. PMC8132692 34035697

[59] López-Trujillo MA, Olivares-Gazca JM, Cantero-Fortiz Y, García-Navarrete YI, Cruz-Mora A, Olivares-Gazca JC, Murrieta-Álvarez I, León-Peña AA, Ruiz-Delgado GJ, Ruiz-Argüelles GJ. Nonalcoholic fatty liver disease and thrombocytopenia III: its association with insulin resistance. Clin Appl Thromb Hemost. 2019;14(25):1076029619888694. 10.1177/1076029619888694 PMC7019400 31840531

[60] Malladi N, Alam MJ, Maulik SK, Banerjee SK. The role of platelets in non-alcoholic fatty liver disease: from pathophysiology to therapeutics. Prostaglandins Other Lipid Mediat. 2023;1(169):106766. 10.1016/j.prostaglandins.2023.106766 37479133

[61] Dalbeni A, Castelli M, Zoncapè M, Minuz P, Sacerdoti D. Platelets in non-alcoholic fatty liver disease. Front Pharmacol. 2022;18(13):842636. 10.3389/fphar.2022.842636 PMC8895200 35250588

[62] Yin H, Shi A, Wu J. Platelet-activating factor promotes the development of non-alcoholic fatty liver disease. Diabetes, metabolic syndrome and obesity: targets and therapy. 2022;1:2003–30. 10.2147/DMSO.S367483 PMC9275506 35837578

[63] Wang S, Zhang C, Zhang G, Yuan Z, Liu Y, Ding L, Sun X, Jia H, Xue F. Association between white blood cell count and non-alcoholic fatty liver disease in urban Han Chinese: a prospective cohort study. BMJ Open. 2016;6(6):e010342. 27251683 10.1136/bmjopen-2015-010342 PMC4893843

[64] Katsiki N, Mikhailidis DP, Mantzoros CS. Non-alcoholic fatty liver disease and dyslipidemia: an update. Metabolism. 2016;65(8):1109–23. 27237577 10.1016/j.metabol.2016.05.003

[65] Martin A, Lang S, Goeser T, Demir M, Steffen HM, Kasper P. Management of dyslipidemia in patients with non-alcoholic fatty liver disease. Curr Atheroscler Rep. 2022;24(7):533–46. 35507279 10.1007/s11883-022-01028-4 PMC9236990

[66] Natarajan Y, Kramer JR, Yu X, Li L, Thrift AP, El-Serag HB, Kanwal F. Risk of cirrhosis and hepatocellular cancer in patients with NAFLD and normal liver enzymes. Hepatology. 2020;72(4):1242–52. 32022277 10.1002/hep.31157 PMC8318072

[67] Kalas MA, Chavez L, Leon M, Taweesedt PT, Surani S. Abnormal liver enzymes: A review for clinicians. World J Hepatol. 2021;13(11):1688. 34904038 10.4254/wjh.v13.i11.1688 PMC8637680

[68] Huang YH, Chan C, Lee HW, Huang C, Chen YJ, Liu PC, Lu SN, Chuang WL, Huang JF, Yu ML, Koshiol J. Influence of nonalcoholic fatty liver disease with increased liver enzyme levels on the risk of cirrhosis and hepatocellular carcinoma. Clin Gastroenterol Hepatol. 2023;21(4):960–9. 35124270 10.1016/j.cgh.2022.01.046 PMC9349477

[69] Iluz-Freundlich D, Zhang M, Uhanova J, Minuk GY. The relative expression of hepatocellular and cholestatic liver enzymes in adult patients with liver disease. Ann Hepatol. 2020;19(2):204–8. 31628070 10.1016/j.aohep.2019.08.004

[70] Sun L, Wang Q, Liu M, Xu G, Yin H, Wang D, Xie F, Jin B, Jin Y, Yang H, Zhou J. Albumin binding function is a novel biomarker for early liver damage and disease progression in non-alcoholic fatty liver disease. Endocrine. 2020;69:294–302. 32399892 10.1007/s12020-020-02319-z

[71] Kawaguchi K, Sakai Y, Terashima T, Shimode T, Seki A, Orita N, Takeshita Y, Shimakami T, Takatori H, Arai K, Kitamura K. Decline in serum albumin concentration is a predictor of serious events in nonalcoholic fatty liver disease. Medicine. 2021;100(31):e26835. 34397849 10.1097/MD.0000000000026835 PMC8341320

[72] Takahashi H, Kawanaka M, Fujii H, Iwaki M, Hayashi H, Toyoda H, Oeda S, Hyogo H, Morishita A, Munekage K, Kawata K. Association of serum albumin levels and long-term prognosis in patients with biopsy-confirmed nonalcoholic fatty liver disease. Nutrients. 2023;15(9):2014. 37432160 10.3390/nu15092014 PMC10180563

[73] Masroor M, Haque Z. HbA1C as a biomarker of non-alcoholic fatty liver disease: comparison with anthropometric parameters. J Clin Transl Hepatol. 2021;9(1):15. 33604251 10.14218/JCTH.2019.00046 PMC7868696

[74] Colosimo S, Miller H, Koutoukidis DA, Marjot T, Tan GD, Harman DJ, Aithal GP, Manousou P, Forlano R, Parker R, Sheridan DA. Glycated haemoglobin is a major predictor of disease severity in patients with NAFLD. Diabetes Res Clin Pract. 2024;1(217):111820. 10.1016/j.diabres.2024.111820 PMC7619438 39147101

[75] Wang JW, Jin CH, Ke JF, Ma YL, Wang YJ, Lu JX, Li MF, Li LX. GA/HbA1c ratio is a simple and practical indicator to evaluate the risk of metabolic dysfunction-associated fatty liver disease in type 2 diabetes: an observational study. Diabetol Metab Syndr. 2022;14(1):167. 36369095 10.1186/s13098-022-00946-2 PMC9652955

[76] Wang TY, Wang RF, Bu ZY, Targher G, Byrne CD, Sun DQ, Zheng MH. Association of metabolic dysfunction-associated fatty liver disease with kidney disease. Nat Rev Nephrol. 2022;18(4):259–68. 35013596 10.1038/s41581-021-00519-y

[77] Hashimoto Y, Hamaguchi M, Okamura T, Nakanishi N, Obora A, Kojima T, Fukui M. Metabolic associated fatty liver disease is a risk factor for chronic kidney disease. J Diab Invest. 2022;13(2):308–16. 10.1111/jdi.13678 PMC8847128 34561962

[78] Platon ML, Stefanescu H, Muresan D, Florea M, Szász ME, Maniu A, Badea R. Noninvasive assessment of liver steatosis using ultrasound methods. Med Ultrason. 2014;16(3):236–45. 25110765 10.11152/mu.2013.2066.163.1mlp

[79] Bozic D, Podrug K, Mikolasevic I, Grgurevic I. Ultrasound methods for the assessment of liver steatosis: a critical appraisal. Diagnostics. 2022;12(10):2287. 36291976 10.3390/diagnostics12102287 PMC9600709

[80] Ballestri S, Nascimbeni F, Lugari S, Lonardo A, Francica G. A critical appraisal of the use of ultrasound in hepatic steatosis. Expert Rev Gastroenterol Hepatol. 2019;13(7):667–81. 31104523 10.1080/17474124.2019.1621164

[81] Jeon SK, Lee JM, Joo I, Park SJ. Quantitative ultrasound radiofrequency data analysis for the assessment of hepatic steatosis in nonalcoholic fatty liver disease using magnetic resonance imaging proton density fat fraction as the reference standard. Korean J Radiol. 2021;22(7):1077. 33739636 10.3348/kjr.2020.1262 PMC8236371

[82] Torres L, Schuch A, Longo L, Valentini BB, Galvão GS, Luchese E, Pinzon C, Bartels R, Álvares-da-Silva MR. New FIB-4 and NFS cutoffs to guide sequential non-invasive assessment of liver fibrosis by magnetic resonance elastography in NAFLD. Ann Hepatol. 2023;28(1):100774. 36280013 10.1016/j.aohep.2022.100774

[83] Han S, Choi M, Lee B, Lee HW, Kang SH, Cho Y, Ahn SB, Jun DW, Lee J, Yoo JJ. Accuracy of noninvasive scoring systems in assessing liver fibrosis in patients with nonalcoholic fatty liver disease: a systematic review and meta-analysis. Gut and Liver. 2022;16(6):952. 35193993 10.5009/gnl210391 PMC9668505

[84] Murayama K, Okada M, Tanaka K, Inadomi C, Yoshioka W, Kubotsu Y, Yada T, Isoda H, Kuwashiro T, Oeda S, Akiyama T. Prediction of nonalcoholic fatty liver disease using noninvasive and non-imaging procedures in Japanese health checkup examinees. Diagnostics. 2021;11(1):132. 33467114 10.3390/diagnostics11010132 PMC7830542

[85] Deprince A, Haas JT, Staels B. Dysregulated lipid metabolism links NAFLD to cardiovascular disease. Mol Metab. 2020;1(42):101092. 10.1016/j.molmet.2020.101092 PMC7600388 33010471

[86] Taheri E, Moslem A, Mousavi-Jarrahi A, Hatami B, Pourhoseingholi MA, Aghdaei HA, Zali MR. Predictors of metabolic-associated fatty liver disease (MAFLD) in adults: a population-based study in Northeastern Iran. Gastroenterol Hepatol from bed to bench. 2021;14(Suppl1):S102. PMC8817755 35154609

[87] Li Y, Chen Y, Tian X, Zhang S, Jiao J. Comparison of clinical characteristics between obese and non-obese patients with nonalcoholic fatty liver disease (NAFLD). Diabetes, Metabolic Syndrome and Obesity. 2021;6:2029–39. 10.2147/DMSO.S304634 PMC8110261 33986604

[88] Keivanlou MH, Amini-Salehi E, Sattari N, Hashemi M, Saberian P, Prabhu SV, Javid M, Mirdamadi A, Heidarzad F, Letafgatkar N, Zare R. Gut microbiota interventions in type 2 diabetes mellitus: An umbrella review of glycemic indices. Diabetes Metab Syndr. 2024;23:103110. 10.1016/j.dsx.2024.103110 39213690

[89] Mahapatro A, Bawna F, Kumar V, Daryagasht AA, Gupta S, Raghuma N, Moghdam SS, Kolla A, Mahapatra SS, Sattari N, Amini-Salehi E. Anti-inflammatory effects of probiotics and synbiotics on patients with non-alcoholic fatty liver disease: an umbrella study on meta-analyses. Clin Nutr ESPEN. 2023;1(57):475–86. 10.1016/j.clnesp.2023.07.087 37739694

